# Supplementary material for: The RNA-binding protein ARPP21 controls dendritic branching by functionally opposing the miRNA it hosts
Source: Nat Commun. 2018 Mar 26;9:1235. doi: 10.1038/s41467-018-03681-3 (PMC5964322; doi:10.1038/s41467-018-03681-3)
Supplement: Supplementary file 1 — Supplementary Information(PDF 28096 kb) [file 41467_2018_3681_MOESM1_ESM.pdf]

Rehfeld et al. Supplementary Information

The RNA-binding protein ARPP21 controls dendritic branching by functionally opposing the miRNA it hosts

Supplementary Figures 1-21

Supplementary Tables 1-5

Supplementary Materials and Methods

Supplementary References

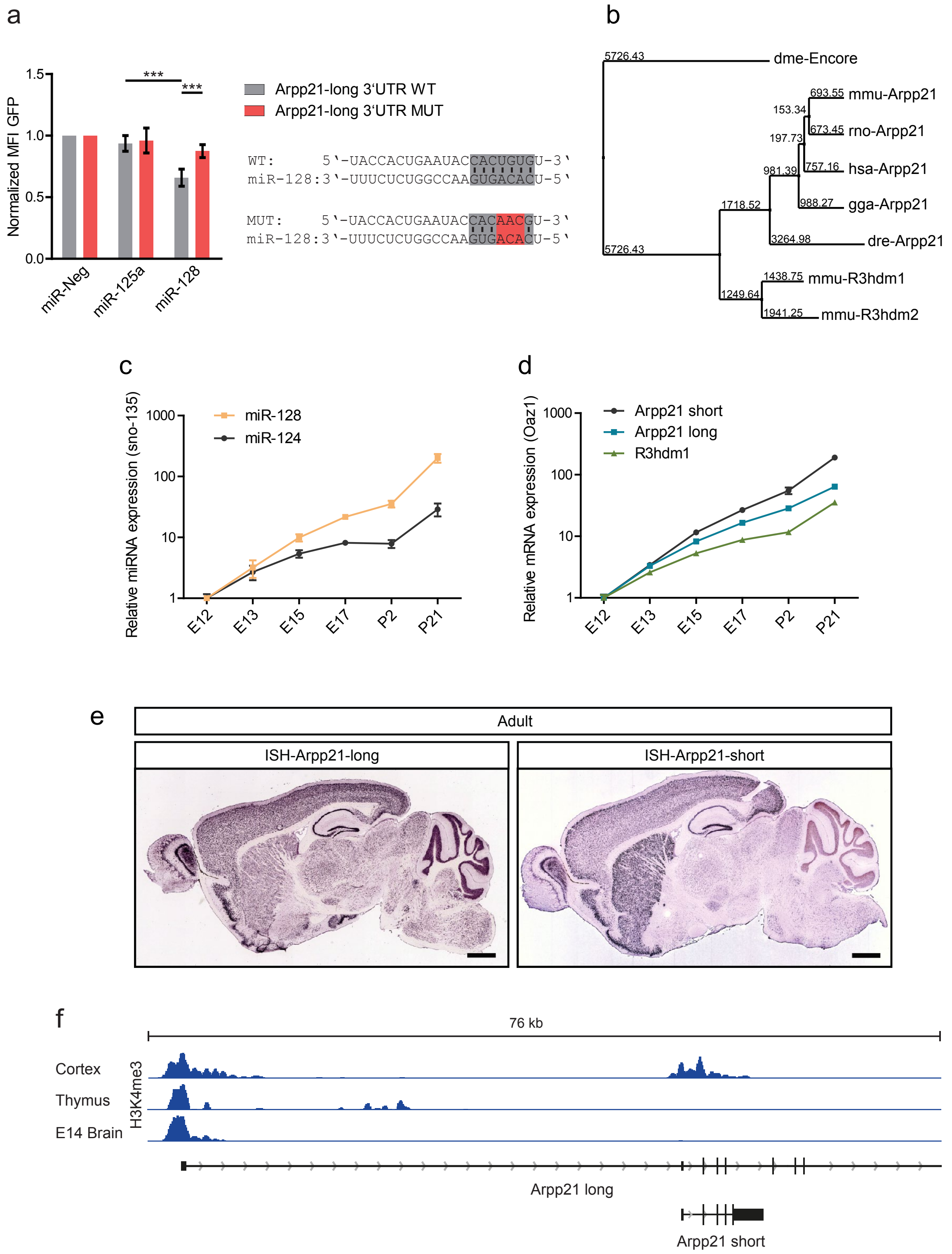

Supplementary Figure 1. miR-128 host gene conservation and expression of miR-128 and its host transcripts during mouse brain development. (**a**) miR-128 negatively regulates its own host transcript *Arpp21*. Transfection of synthetic miR-128 into 293T cells inhibits the expression of a GFP-reporter construct carrying the long *Arpp21* isoform 3'UTR. The mean GFP fluorescence intensity is normalized to a control transfection with a synthetic negative control

RNA. The 3’UTR of *Arpp21* does not possess any predicted binding site for miR-125 and transfection of synthetic miR-125 does not decrease the expression of the *Arpp21* 3’UTR GFP reporter compared to the negative control. Mutation of the 7mer miR-128 binding site in the *Arpp21* 3’UTR significantly rescues the observed targeting effect upon transfection of the miR-128 mimic. (***) $p < 0.001$, 2-way ANOVA with Bonferroni posttest, bar graph shows mean ± s.d., n = 3. (**b**) Phylogenetic tree showing the average distance (PAM250) of ARPP21 protein from different vertebrate species, murine R3HDM1, murine R3HDM2 and ENCORE protein. Generated with Jalview 2.1 [1]. (**c**) Relative expression of miR-128 and miR-124 during brain development quantified by TaqMan miRNA qRT-PCR. Expression is normalized to the housekeeping control sno-135 and to values at E12. n = 4 biological replicates. (**d**) qRT-PCR analysis of miR-128 host gene expression during brain development. Expression is normalized to the housekeeping control *Oaz1* and to values at E12. Data are shown as mean ± s.d. of n = 4 biological replicates. *Arpp21* short and long splice variants are described in Fig. 1. (**e**) mRNA *in situ* analysis of the long and short *Arpp21* splice variants. The short *Arpp21* isoform shows an enriched *in situ* signal in the cortex and the striatum (right panel). The long *Arpp21* splice variant shows a more ubiquitous expression pattern throughout the brain (left panel). Data from the Allen Mouse Brain Atlas [2] experiments: #79677353 (Arpp21-long) and #68148752 (Arpp21-short). (**f**) ENCODE H3K4me3 ChIP-Seq analysis at the *Arpp21* promoter in samples from mouse adult cortex, thymus and E14 whole brain. Two distinct H3K4me3 peaks are visible at the *Arpp21* locus: One distal peak, coinciding with the expression pattern of the long-*Arpp21* isoform and another proximal peak, closer to the first coding exon, coinciding with expression of the short *Arpp21* isoform.

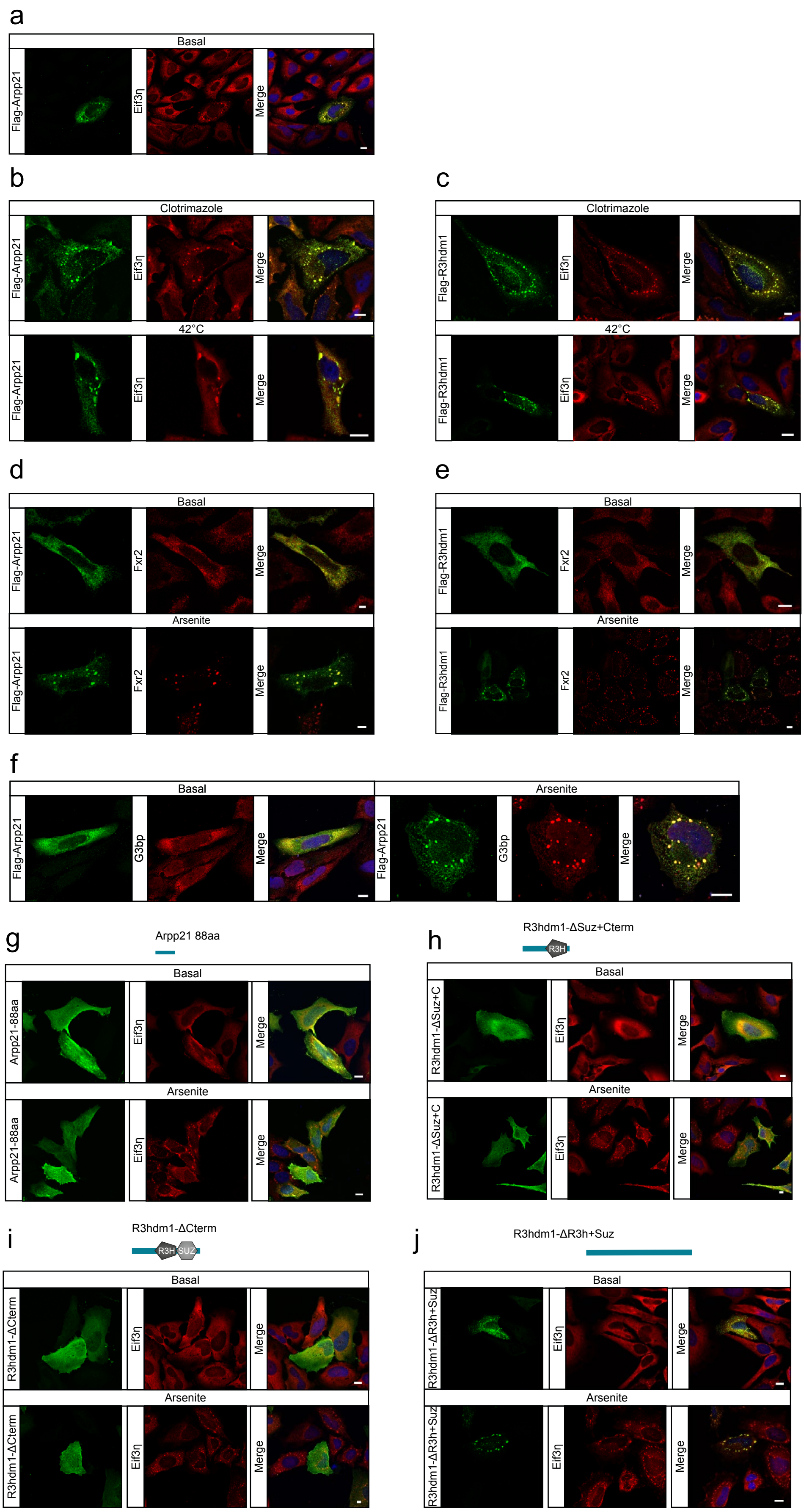
a
Basal
Flag-Arpp21
Eif3η
Merge
b
Clotrimazole
42°C
Flag-Arpp21
Eif3η
Merge
c
Clotrimazole
42°C
Flag-R3hdm1
Eif3η
Merge
d
Basal
Arsenite
Flag-Arpp21
Fxr2
Merge
e
Basal
Arsenite
Flag-R3hdm1
Fxr2
Merge
f
Basal
Arsenite
Flag-Arpp21
G3bp
Merge
g
Arpp21 88aa
Basal
Arsenite
Arpp21-88aa
Eif3η
Merge
h
R3hdm1-ΔSuz+Cterm
R3H
Basal
Arsenite
R3hdm1-ΔSuz+C
Eif3η
Merge
i
R3hdm1-ΔCterm
R3H
SUZ
Basal
Arsenite
R3hdm1-ΔCterm
Eif3η
Merge
j
R3hdm1-ΔR3h+Suz
Basal
Arsenite
R3hdm1-ΔR3h+Suz
Eif3η
Merge

Supplementary Figure 2. Different stimuli and stress granule markers confirm the miR-128 host proteins' localization to stress granules. (**a**) After transfection of a FLAG-tagged full-length ARPP21 expression construct (green), occasional cells demonstrated colocalization to EIF3η^{+} aggregates (red) under basal conditions. EIF3η was predominantly diffusely localized in non-transfected cells. (**b**,**c**) After transfection with epitope-tagged expression constructs, cells treated with clotrimazole (upper panel) or heat-shock (lower panel) show ARPP21 and R3HDM1 localization to granules that are positive for the stress granule marker protein EIF3η. (**d,e**) After transfection with epitope-tagged expression constructs, ARPP21, R3HDM1 and the endogenous stress granule marker protein FXR2 show diffuse, granular, and exclusively cytoplasmic staining under basal conditions (upper panels). Upon arsenite stimulation, ARPP21 and R3HDM1 colocalize with FXR2 in cytosolic stress granules (lower panels). (**f**) Transfected ARPP21 colocalizes with stress granule marker G3BP upon arsenite treatment. (**g**) The short, 88 aa, ARPP21 protein variant (green) shows diffuse nuclear and cytoplasmic staining under basal conditions (upper panel) which does not appreciably change upon cellular stress (lower panel) compared to the SG marker EIF3η. (**h-j**) Cytoplasmic localization of R3HDM1 deletion mutants was determined in response to arsenite treatment in transfection experiments. R3HDM1 deletion mutants lacking either the SUZ domain and C-terminus (**h**) or only the C-terminus (**i**) show primarily cytoplasmic localization under basal conditions (upper panels) and do not exhibit stress granule localization upon arsenite treatment (lower panels). (**j**) R3HDM1 deleted for the N-terminus and the R3H and SUZ domains exhibits cytosolic, granular staining (upper panel) and relocates to stress granules upon arsenite stimulation (lower panel). Scale bar in all subfigures: 10 µm.

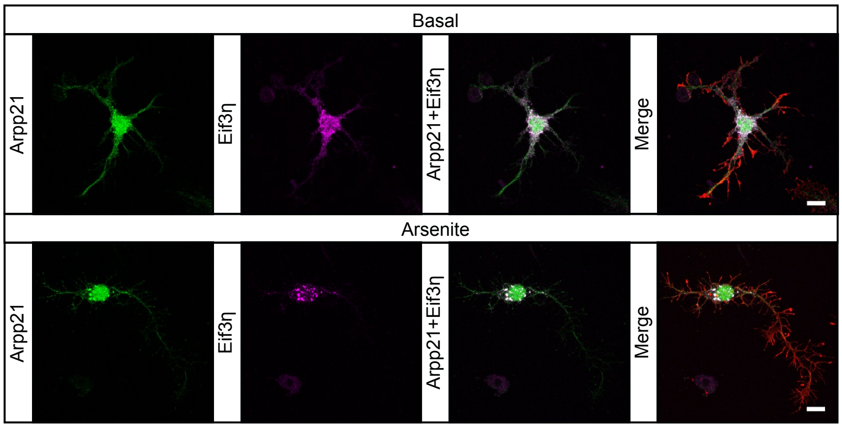

Supplementary Figure 3. Endogenous ARPP21 localization in primary cortical neurons under basal and stress conditions. Under basal conditions ARPP21 (green) is primarily localized to the neuronal cell body and proximal dendrites. Upon arsenite application ARPP21 is present in aggregates that co-stain for the stress granule marker EIF3η (magenta). The merged image shows ARPP21 and EIF3η staining together with the cytoskeletal actin stain phalloidin (red). Scale bar: 10μm.

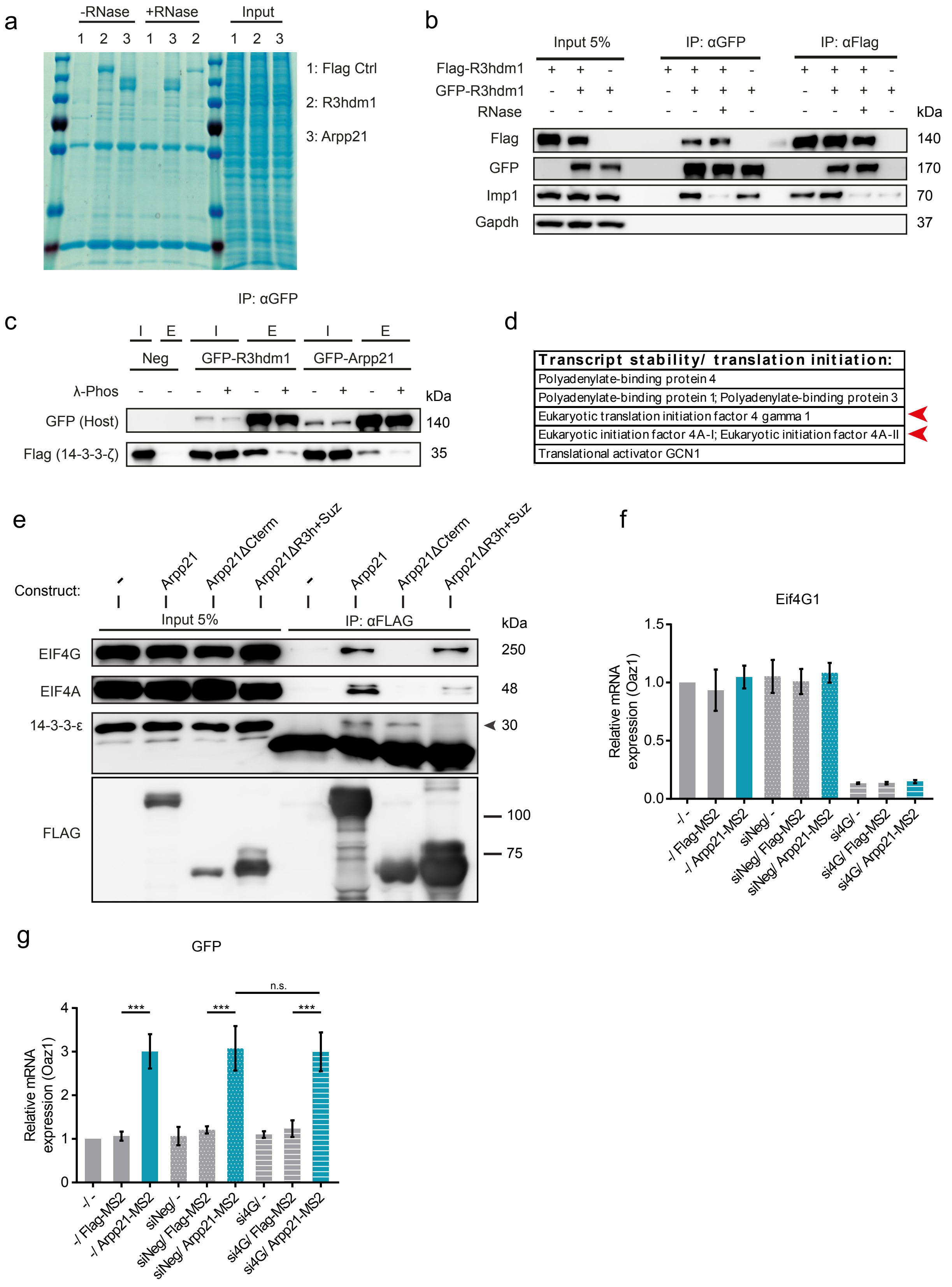

Supplementary Figure 4. Validation experiments for interaction partners identified in the ARPP21 and R3HDM1 MS-screen. **(a)** Eluates of host-protein anti-FLAG IPs used for MS were analyzed by SDS-PAGE and staining with PageBlue (ThermoScientific). IPs from empty FLAG

vector control (1), FLAG-R3HDM1 (2) and FLAG-ARPP21 (3) transfected 293T cells are shown. The bait and several additional co-purifying proteins are visible in immunoprecipitates of ARPP21 and R3HDM1. (**b**) Immunoprecipitation of FLAG-R3HDM1 retrieves co-expressed GFP-R3HDM1 and vice versa. This homomeric interaction is preserved after RNase treatment. The interaction of R3HDM1 with IMP1 is RNA-dependent and serves as a positive control for the RNase treatment. The blot was probed for GAPDH as a negative control. (**c**) 14-3-3-ζ is the most enriched 14-3-3-class protein identified in the MS host protein interaction screen. GFP-tagged ARPP21 and R3HDM1 each co-immunoprecipitate co-transfected FLAG-14-3-3-ζ upon IP with anti-GFP antibody. These interactions are sensitive to lysate dephosphorylation by pretreatment with λ-phosphatase. I stands for input, E for eluate. (**d**) List of known regulators of mRNA stability and translation identified in the MS screen. (**e**) Co-IP of ARPP21 deletion series to determine the region interacting with EIF4G and EIF4A. The C-terminal domain of ARPP21 is necessary and sufficient to co-immunoprecipitate EIF4G and EIF4A (compare Lanes 6 and 8 with Lane 7), but is dispensable for the interaction of ARPP21 with 14-3-3-ε (compare Lanes 6 and 7 with Lane 8). Detection of individual FLAG-tagged deletion proteins is shown in the bottom panel. (**f,g**) RNA levels of *Eif4g1* and the *GFP* reporter in the ARPP21 tethering assay, normalized to the standard mRNA *Oaz1*. (**f**) siRNA against *Eif4g1* (si4G) leads to strongly reduced *Eif4g1* mRNA level compared to control (siNeg) for all co-transfections. (**g**) ARPP21 tethering (Arpp21-MS2) leads to a significant increase in *GFP* reporter mRNA expression compared to mock transfection (-) or negative control (Flag-MS2) as determined by qRT-PCR normalized to the standard mRNA *Oaz1*. This increase is unaffected by knockdown of *Eif4g1*. (***) $p < 0.001$, 2-way ANOVA with Bonferroni post-test, data are shown as mean ± s.d., $n = 3$.

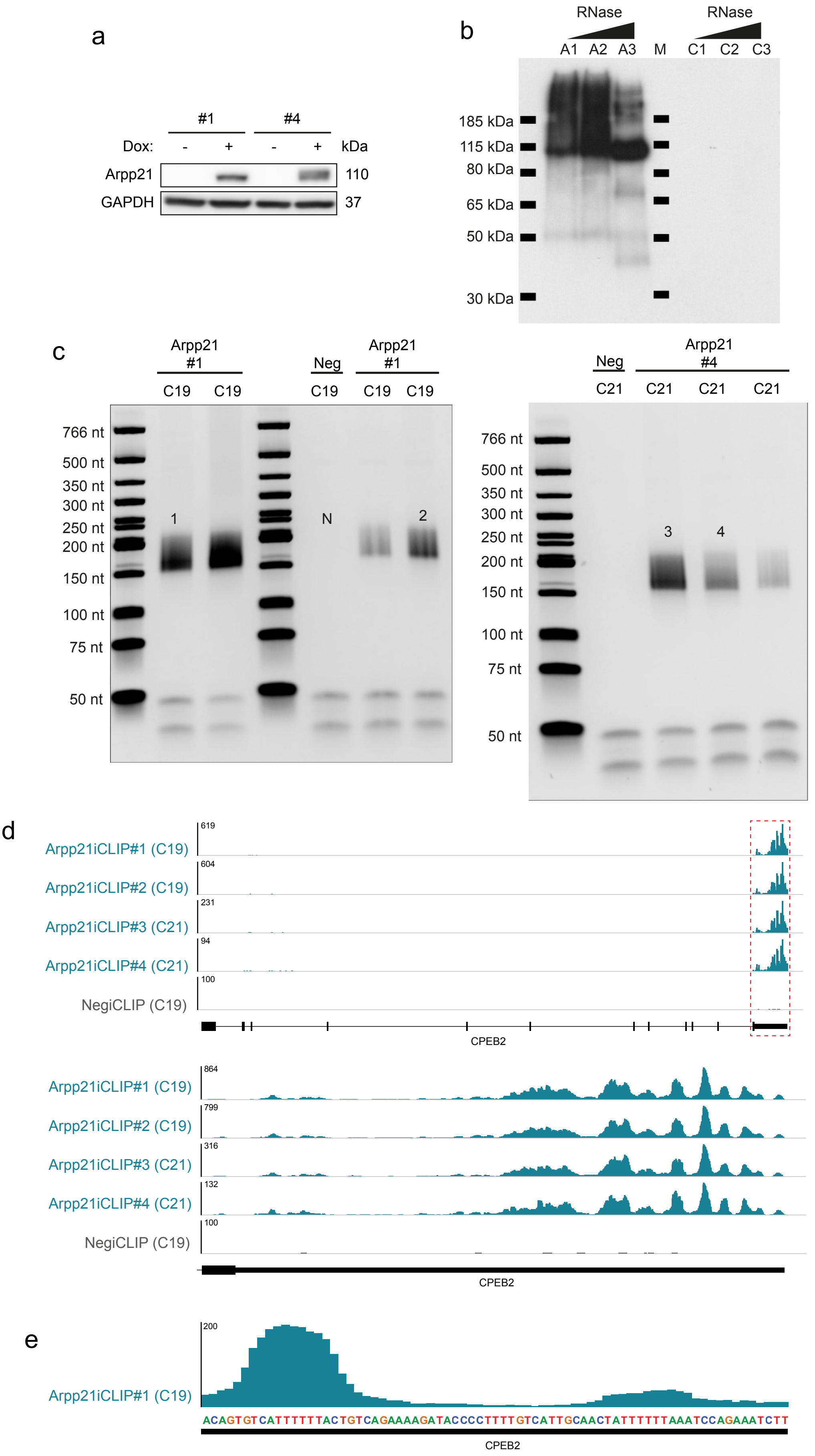
a
#1
#4
Dox:
-
+
-
+
kDa
Arpp21
110
GAPDH
37
b
RNase
A1 A2 A3 M
RNase
C1 C2 C3
185 kDa
115 kDa
80 kDa
65 kDa
50 kDa
30 kDa
c
Arpp21
#1
C19 C19
Neg
C19
Arpp21
#1
C19 C19
766 nt
500 nt
350 nt
300 nt
250 nt
200 nt
150 nt
100 nt
75 nt
50 nt
1
N
2
Neg
C21
Arpp21
#4
C21 C21 C21
3
4
d
Arpp21iCLIP#1 (C19)
Arpp21iCLIP#2 (C19)
Arpp21iCLIP#3 (C21)
Arpp21iCLIP#4 (C21)
NegiCLIP (C19)
CPEB2
e
Arpp21iCLIP#1 (C19)
ACAGTGTCATTTTTTACTGTCAGAAAAGATACCCCTTTTGTCATTGCAACTATTTTTTAAATCCAGAAATCTT
CPEB2

Supplementary Figure 5. iCLIP quality control experiments. (**a**) Immunoblot against ARPP21 in two independent clones of 293-TREx-HA-Arpp21 (iARPP21) cells untreated (-) or treated (+) with 1 µg/ml doxycycline. Moderate ARPP21 expression is visible upon Doxycycline addition. (**b**) CLIP experiments reveal that ARPP21 binds RNA and show the specificity of the HA-immunoprecipitation. CLIP with HA-antibody from iARPP21 cells shows ARPP21-RNA complexes that are trimmed by treatment with escalating amounts of RNase I prior to labeling. Dilutions of RNase I stock (100 U/µl) were: A1=1:200, A2=1:100, A3=1:10. An RNase dilution of 1:100 was used for preparative iCLIP experiments. Radioactive signal is absent in the CLIP experiment after IP with HA-antibody from control 293-TREx cells not expressing ARPP21. (**c**) 10% TBE-PAGE of a fraction of the final, amplified ARPP21-iCLIP cDNA libraries. C19 and C21 refer to the respective number of PCR cycles (19 and 21) used for cDNA library amplification. ARPP21 iCLIP cDNA libraries 1,2,3,4 and one negative control library generated from cells that do not express ARPP21 but underwent the same treatment were used for deep sequencing. (**d**) iCLIP signal of the four independent ARPP21 iCLIP experiments and the negative control iCLIP at the CPEB2 locus. The lower panel shows a magnification of the CPEB2 3'UTR. All four ARPP21 iCLIP experiments show congruent binding patterns, demonstrating the degree of experimental reproducibility. The control iCLIP shows negligible background signal compared to the ARPP21 iCLIP. (**e**) Not all uridine-rich sequences are bound by ARPP21. Differential binding to two adjacent uridine 6mers present in the CPEB2 3'UTR are shown (numerical scale on the left refers to number of unique crosslinking events/nucleotide). Sequence context and local secondary structure are likely to influence ARPP21 binding to poly-U motifs.

a

#	GO term: biological process	Adj. p-value
1	GO:0098609 cell-cell adhesion	2.00E-09
2	GO:0016032 viral process	1.73E-07
3	GO:0051301 cell division	3.13E-06
4	GO:0000398 mRNA splicing, via spliceosome	3.29E-05
5	GO:0006397 mRNA processing	4.12E-04

b

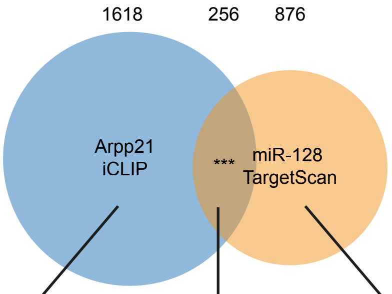

c

#	PathwayName	Adj. p-value
1	Protein processing in ER	1.29E-07
2	Cell cycle	1.55E-06
3	mRNA surveillance pathway	2.00E-05
4	Oocyte meiosis	6.14E-05
5	Vibrio cholerae infection	0.0001
6	Ubiquitin mediated proteolysis	0.0001
7	Spliceosome	0.0004
8	Chronic myeloid leukemia	0.0005
9	**Neurotrophin signaling pathway**	0.0009
10	RNA transport	0.0009
...	...	...
22	**TGF-beta signaling pathway**	0.0103

d

#	PathwayName	Adj. p-value
1	**Neurotrophin signaling pathway**	0.0067
2	Axon guidance	0.0069
3	Focal adhesion	0.0069
4	Insulin signaling pathway	0.0069
5	Pathways in cancer	0.0092
6	**TGF-beta signaling pathway**	0.0092
7	MAPK signaling pathway	0.0099
8	ErbB signaling pathway	0.0276
9	Colorectal cancer	0.0357
10	Renal cell carcinoma	0.0357

e

#	PathwayName	Adj. p-value
1	**TGF-beta signaling pathway**	0.0007
2	Chronic myeloid leukemia	0.0102
3	Colorectal cancer	0.0187
4	**Neurotrophin signaling pathway**	0.0187
5	Sphingolipid metabolism	0.0218
6	MAPK signaling pathway	0.0249
7	Endometrial cancer	0.031
8	mRNA surveillance pathway	0.031
9	Glycerophospholipid metabolism	0.031
10	Prostate cancer	0.0328

Supplementary Figure 6. Comparative GO and KEGG pathway analysis for mRNAs identified in the ARPP21 iCLIP and for predicted targets of miR-128. (**a**) Top five biological process GO terms for ARPP21 target mRNAs identified by iCLIP. (**b**) Venn diagram showing the overlap of mRNAs identified in the ARPP21 iCLIP and mRNAs with TargetScan 7.1 predictions for conserved miR-128 binding sites that are expressed in TREx cells. Hypergeometric test of population overlap. (***) $p < 4.7*10^{-41}$. (**c**,**d**,**e**) KEGG pathway enrichment analysis of genes identified in the ARPP21 iCLIP (**c**), TargetScan miR-128 predictions (**d**), and their intersection (**e**). KEGG categories known to be regulated by miR-128 based on published reports are marked in

red. Pathways that are present in all three populations are marked in bold. The complete list of genes can be found in Supplemental Table 6.

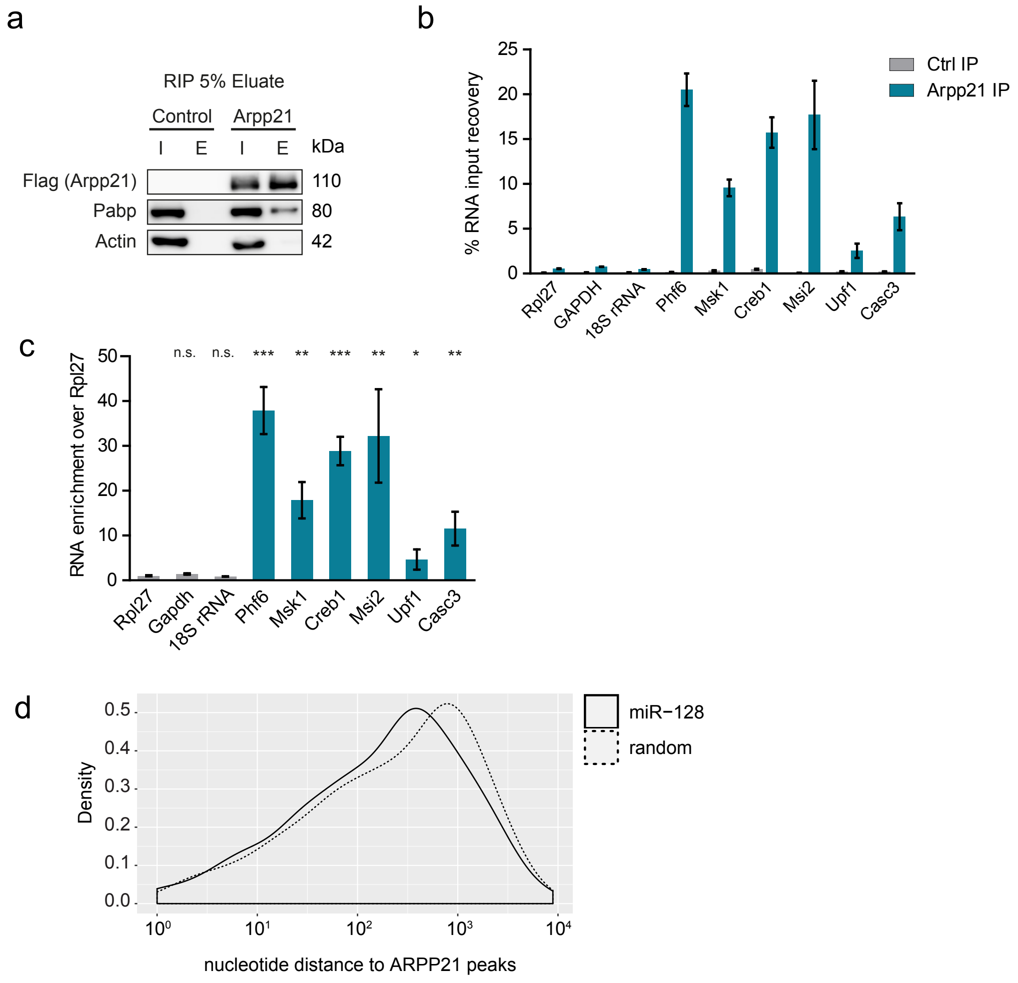

Supplementary Figure 7. RNA-immunoprecipitation (RIP) of FLAG-ARPP21 from 293T cells and distance of ARPP21 crosslinking-sites to miR-128 binding sites. Transcripts identified in the ARPP21 iCLIP are strongly enriched upon ARPP21 RIP. (**a**) SDS-PAGE of input (I) and eluate (E) shows efficient enrichment of FLAG-ARPP21 upon FLAG-IP. Note that the polyA-binding protein PABP is co-immunoprecipitated together with ARPP21, but is absent in the eluate from an immunoprecipitation of control cells not expressing ARPP21. (**b**) qRT-PCR quantification of RNA recovery upon control and ARPP21 immunoprecipitation for a panel of ARPP21 target and non-target transcripts from the ARPP21-iCLIP dataset, expressed as per cent of input present in the eluate. (**c**) The same dataset as in **b**, expressed as eluate over input enrichment upon ARPP21 RIP normalized to the *Rpl27* mRNA. Transcripts identified in the ARPP21 iCLIP experiment (*Phf6*, *Msk1*, *Creb1*, *Msi2*, *Upf1*, *Casc3*) are significantly enriched in the ARPP21 RIP com-

pared to *Rpl27*. Transcripts not identified in the iCLIP (*Gapdh*, *18S* rRNA) are not significantly enriched over *Rpl27*. (***) $p < 0.001$, (**) $p < 0.01$, (*) $p < 0.05$, Student's t-test, mean ± s.d., n = 3. (**d**) Distance between ARPP21 iCLIP peaks and the next conserved miR-128 binding site defined by TargetScan (continuous line). Dotted line shows the distance between ARPP21 peaks and miR-128 binding sites after computationally randomizing miR-128 site distribution. Median distance miR-128: 208 nucleotides. Median distance random: 227 nucleotides. Two-tailed Mann–Whitney U-test, n = 307, p-value = 0.18.

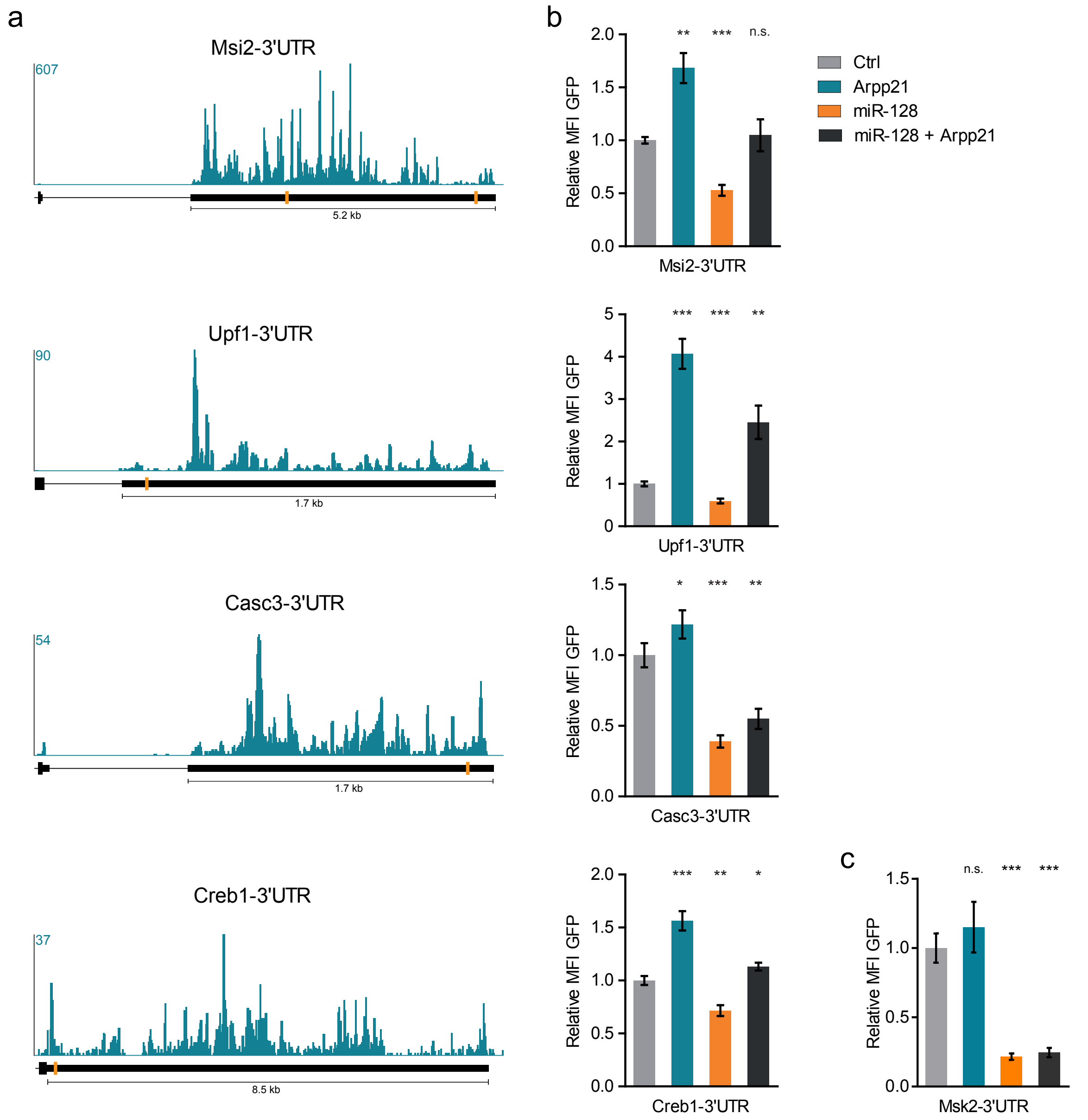

Supplementary Figure 8. iCLIP validation: representative miR-128 target mRNAs and their regulation by ARPP21. (**a**) iCLIP signal (turquoise) of the 3'UTRs of one predicted (*Msi2*) and

three published target mRNAs of miR-128 (*Upf1*[3], *Casc3*[3], *Creb1*[4]). Conserved TargetScan predictions for miR-128 binding sites within the 3'UTR are displayed in orange. (**b**) The 3'UTRs of *Msi2*, *Upf1*, *Casc3* and *Creb1* were cloned in a GFP reporter to assess the direct regulatory effects of miR-128 and ARPP21 on these transcripts. The fluorescence of all 3'UTR reporter constructs is significantly inhibited by miR-128 overexpression, whereas ARPP21 expression leads to a significantly increased GFP reporter fluorescence compared to control. (***) $p < 0.001$, (**) $p < 0.01$, (*) $p < 0.05$, Student's t-test. Data are shown as mean ± s.d., n = 3. (**c**) The expression of a GFP-reporter construct carrying the 3'UTR of the miR-128 target transcript *Msk2* is inhibited by miR-128 transfection but is not altered by ARPP21 overexpression. *Msk2* was expressed in the TREx cell line but not identified as an ARPP21 target mRNA in the iCLIP (see Supplementary Table 6) and serves as a negative control. (***) $p < 0.001$ Student's t-test. Mean ± s.d. of n = 3.

a mmu-Upf1-3'UTR

```
GGTAGCGGAGGAGCAAGAGCTAAGCTATGTGGCTTAGTCTATCAGCATCTTATTCTGGGTAATAAAAA
ATAAAAATAAATGGATACCTGTTTTCCACTGCTAAAACTGAAGCACCACTGTGTGAGCAGCCGAGGAG
AGGAGAGGAAAGAGGAGCGAGAGCGAGCAGAGAGCGGCCAGGGAGGACGACAGAGCGGAGCGCCGAGG
AGCGGGCGCCCCCTGTGGGCGGCGAGAGGAGAGAGGCCCGCACCGCTGCGAGGCCGGCCCAGCGCCGT
GCCCGCCAGAGGGAGAGGCCTCGCCAGGACCGGCCCCGCTGTTCTTTTTCTTTGTTTCTCGTGATTGA
GGGGCTACGTTTTAGCAGGACGAACTTCGCGTTTCTGTGCCCAAGCGGGCGGCCAGGAGCATCAGTGC
GCGGTCCCAGCGCTAAGGGGTTTCATTTAAAGAAAATACGGTGTTTGGGGTTTTTCTGGTTTTTCTTT
TTTTTTTCTTTTTTTTGGTTTTTTTTTTTTGTTTGTTTGTTTGGTTTTTTTTTTTTTTTTCTTTTTGGTT
TTCCTTTCCTCCCCCCTACCCCCTTCAAAGATTCTTTCAAAGGAGTATGGGAAGTACTTGGATCGGTT
TGTCTCTAGAGACCTGACTGTTAAACCTGAGAGATGCGAGAAGCTTCCGGGAAAGGCAGCGCTGAGAA
GCCTGAGCCCCGCAGCCTGGGATCACCGCCTGCACCGCAGCCGGAGGATTTGTTTTTGAGTTTGAGCT
TCAGAGGCCACAGGCGAACTCGTGAGCACGGCCTGCGGGTTCTTCCCCATCGAGGGTGGAGGCCTCAT
TGCTGGGCGCCCTGCCCCGGACCCCCACCTCCCGCGGCAGCCACCCAACCCCTACCCAGGCACACCTG
CGCCAACGCAATGGAACTTAATTTTTAGTCAAATTTGTTTTAAGCTTTTTGTCACCAATAAAACATG
```

b c

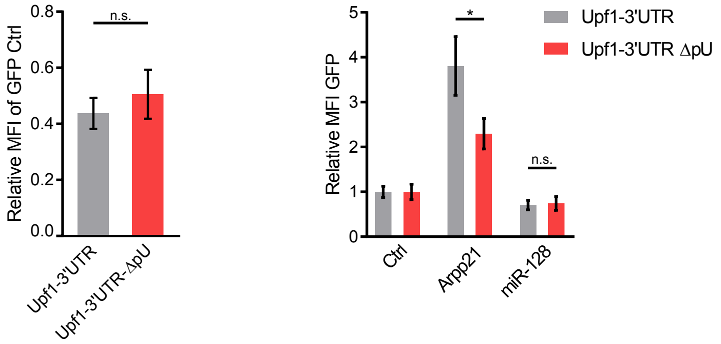

Supplementary Figure 9. Deletion of a poly-uridine motif in the *Upf1*-3'UTR reporter diminishes the response to ARPP21. (**a**) DNA sequence of the murine *Upf1*-3'UTR cloned into the GFP reporter. The conserved miR-128 seed sequence is marked in orange. The poly-uri-

dine/thymidine stretch deleted in the ΔpU reporter is marked in grey. (**b**) The *Upf1*-3'UTR and *Upf1*-3'UTR-ΔpU reporters show comparable GFP reporter fluorescence. (**c**) Upon deletion of the poly-uridine stretch from the *Upf1*-3'UTR reporter, the ability of ARPP21 to increase GFP reporter expression is significantly reduced, without affecting repression by ectopic miR-128. (*) $p < 0.05$, 2-way ANOVA with Bonferroni post-test, bar graphs show mean ± s.d., n = 3.

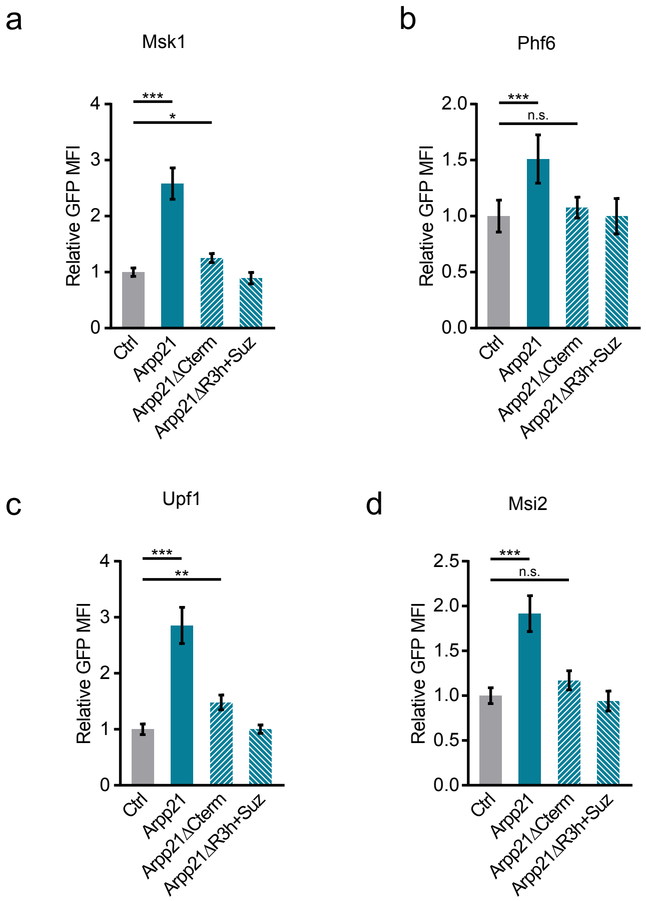

Supplementary Figure 10. 3'UTR sensor assays with ARPP21 deletion constructs. Full-length ARPP21, ARPP21-ΔCterm and ARPP21-ΔR3h+Suz constructs were co-transfected with 3'UTR-reporters derived from *Msk1* (**a**), *Phf6* (**b**), *Upf1* (**c**) and *Msi2* (**d**). Each reporter is responsive to full-length ARPP21. Deletion of the RNA-binding R3H and SUZ domains (ARPP21-ΔR3h+Suz) eliminates transactivation activity for all reporters. Deletion of the C-terminal transactivation domain (ARPP21-ΔCterm) reduces the stimulation for all reporters, although residual activity on the *Msk1* and *Upf1* reporters remains. (***) $p < 0.001$, (**) $p < 0.01$, (*) $p < 0.05$, Student's t-test, bar graphs show mean ± s.d., n = 3.

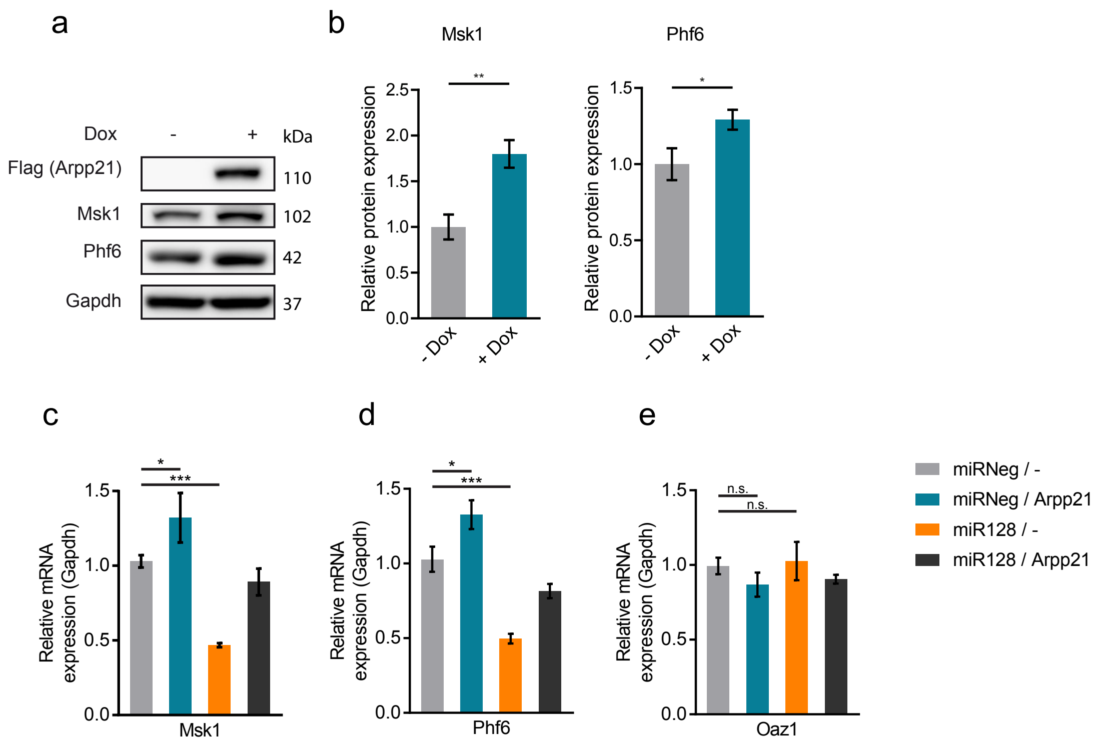

Supplementary Figure 11. Endogenous protein and mRNA expression of *Arpp21* and miR-128 target genes. (**a**) Western Blot of iArpp21 cells stimulated with doxycycline. ARPP21 protein expression is induced and results in increased levels of MSK1 and PHF6 protein. (**b**) Quantification of MSK1 and PHF6 protein expression normalized to GAPDH upon doxycycline stimulation from three independent clones. MSK1 and PHF6 protein expression is significantly increased upon the induction of ARPP21 expression by doxycycline. (**) $p < 0.01$, (*) $p < 0.05$, Student's t-test, bar graphs show mean ± s.d., n = 3 independent clones. mRNA expression of *Msk1* (**c**), *Phf6* (**d**) and *Oaz1* (**e**) in 293T cells upon ARPP21 and/or miR-128 overexpression. (**c**,**d**) *Msk1* and *Phf6* mRNA levels are significantly increased upon ARPP21 expression. miR-128 transfection leads to significantly reduced mRNA expression. (***) $p < 0.001$, (*) $p < 0.05$, Student's t-test, bar graphs show mean ± s.d., n = 3. (**e**) *Oaz1* serves as a negative control and is neither affected by miR-128 nor by ARPP21.

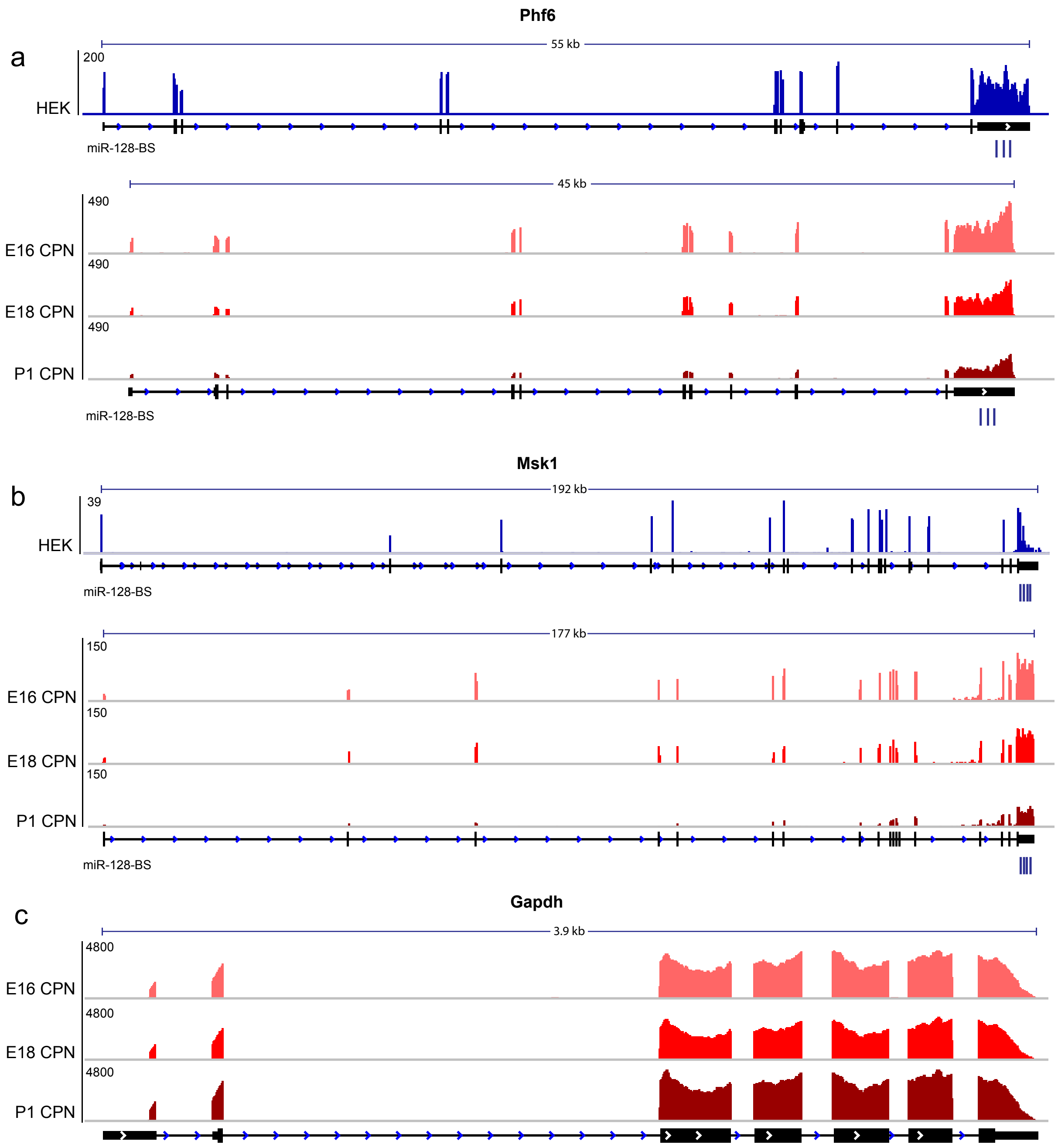

Supplementary Figure 12. Comparison of *Phf6* and *Msk1* transcripts in HEK cells and cortical neurons. mRNA expression data for **(a)** *Phf6* and **(b)** *Msk1* in HEK-293 TREx (HEK) and mouse callosal projection neurons (CPN) at embryonic days (E) 16, 18 and postnatal day (P) 1. Both genes show comparable splicing patterns and 3'UTR coverage. All miR-128 binding sites in *Msk1* and *Phf6* transcripts are present in HEK cells and in cortical neurons in vivo. Note the decrease in *Msk1* and *Phf6* expression from E16 to P1. This decline is reciprocal to the strong increase in miR-128 expression during these time points (see Supplementary Fig. 1c). **(c)** Unlike *Msk1* and *Phf6*, *Gapdh* expression remains stable from E16 to P1. HEK expression data-set from [5] and mouse brain expression datasets from [6].

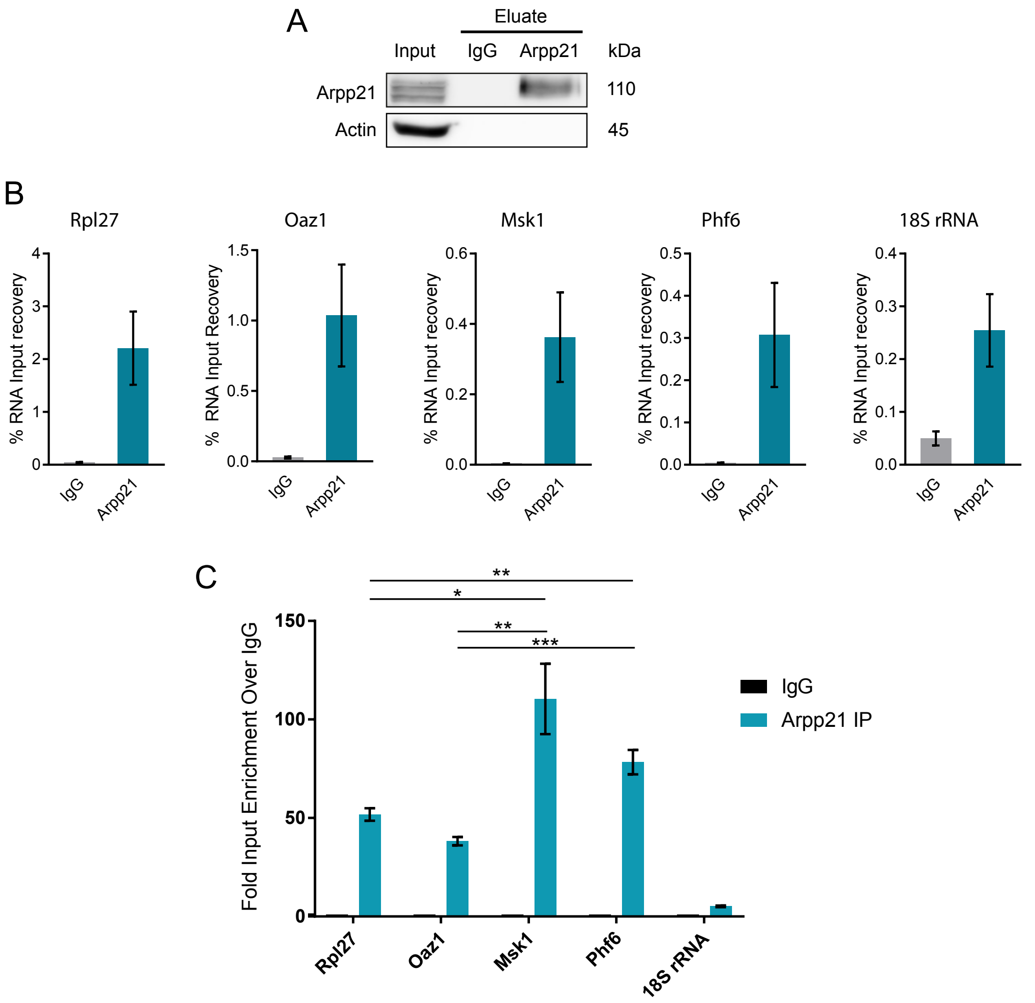

Supplementary Figure 13. RNA-immunoprecipitation of endogenous ARPP21 recovers *Msk1* and *Phf6* transcripts from mouse cortex. (a) Western Blot analysis shows efficient immunoprecipitation of endogenous ARPP21 from E17 mouse cortex tissue. 1% of input lysate and 10% of eluate were loaded on the gel. Actin serves as a negative control and is not immunoprecipitated. **(b)** RNA recovery is high upon ARPP21 immunoprecipitation compared to IgG control immunoprecipitation, consistent with RNA association of ARPP21 *in vivo*. **(c)** To determine the specificity of ARPP21-RNA interactions, the relative enrichment of the target mRNAs identified by iCLIP, *Msk1* and *Phf6*, in ARPP21 immunoprecipitates was calculated and compared to the *Arpp21* non-targets *Rpl27* and *Oaz1*. (***) $p < 0.001$, (**) $p < 0.01$, (*) $p < 0.05$, Student's t-test. Bar graphs show mean ± s.e.m., n = 4 biological replicates.

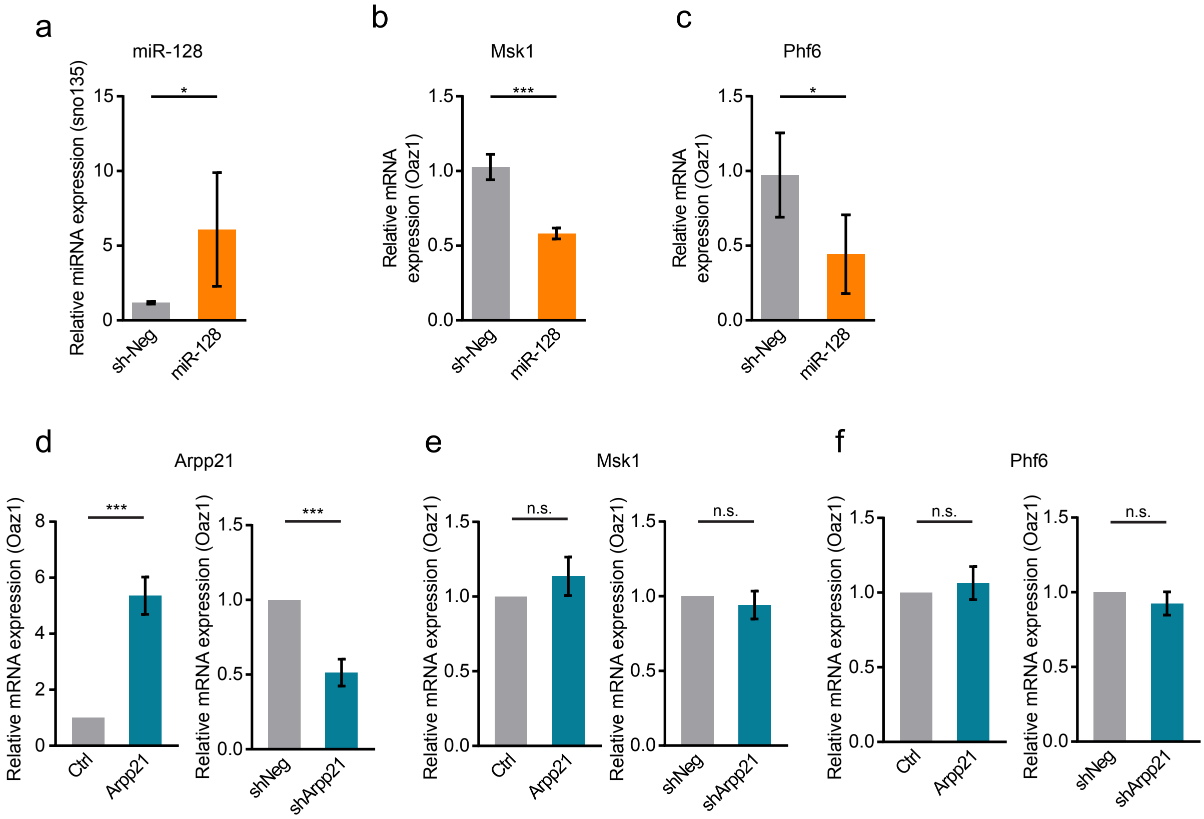

Supplementary Figure 14. Regulation of miR-128 and Arpp21 target mRNAs in primary cortical neurons. (**a**) Lentivirus-based miR-128 overexpression in primary cortical neurons leads to a significant, approximately 5-fold increase in mature miR-128 miRNA as determined by TaqMan qRT-PCR using sno135 for normalization. *Msk1* (**b**) and *Phf6* (**c**) transcript levels are significantly downregulated upon lentivirus-mediated miR-128 overexpression in primary cortical neurons. A negative control hairpin (sh-Neg) expressed from the same viral backbone served as control. (***) $p < 0.001$, (**) $p < 0.01$, (*) $p < 0.05$, Student's t-test. Bar graphs show mean ± s.d., n = 4 biological replicates. (**d**) Lentivirus-based ARPP21 overexpression (left panel) and knockdown (right panel) verification by qRT-PCR in primary cortical neurons. *Msk1* (**e**) and *Phf6* (**f**) mRNA levels are not significantly changed after ARPP21 overexpression or knockdown in primary cortical neurons, in contrast to protein levels (see Fig. 6). Data are presented as mean ± s.d., n = 6 biological replicates.

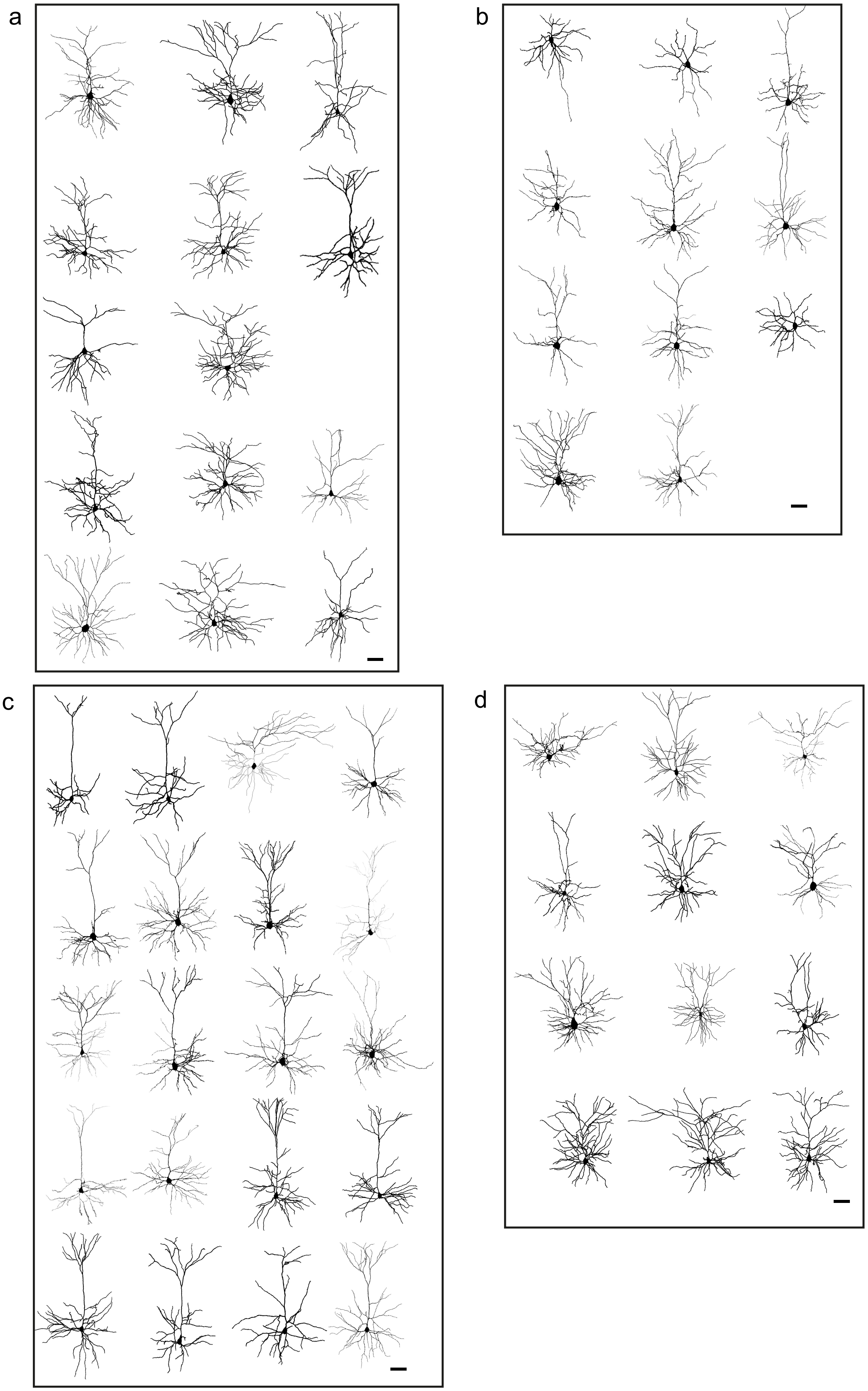
a
b
c
d

Supplementary Figure 15. Complete set of cortical neuron reconstructions from *in vivo* ARPP21 gain- and loss-of-function experiments. (**a**) Negative control shRNA expressing neurons. (**b**) Arpp21-shRNA expressing neurons. (**c**) NeuroD1::GFP control neurons. (**d**) NeuroD1::Arpp21-IRES-GFP neurons. Scale bar: 50µm.

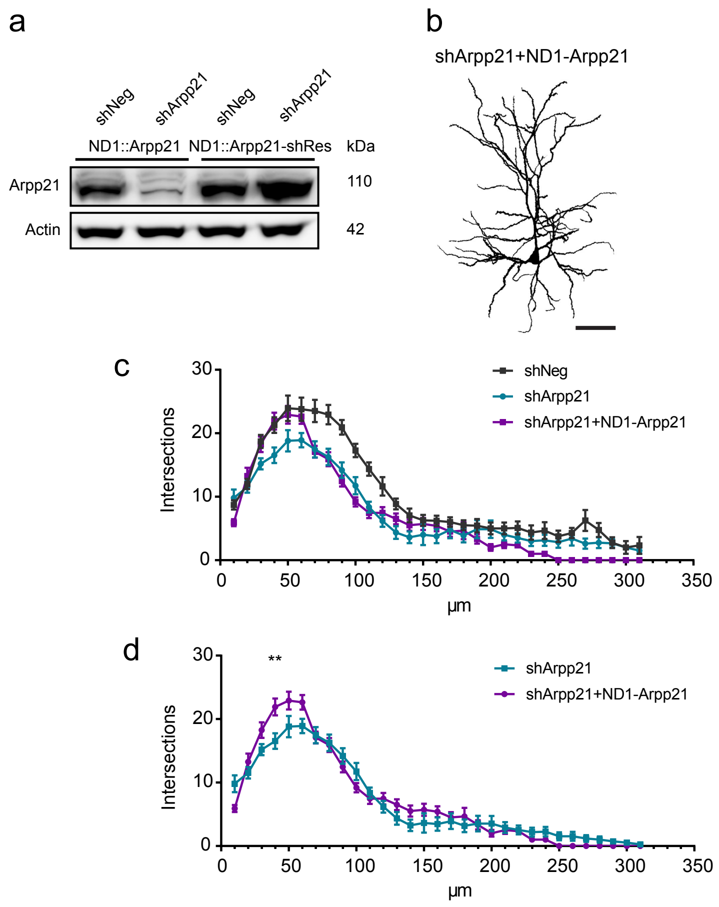

Supplementary Figure 16. Rescue of the *Arpp21* loss-of-function dendritic phenotype by co-electroporation of an shRNA-resistant form of *Arpp21*. (**a**) Verification of the shRNA-resistant mutant ARPP21 mutant. Cells were co-transfected with either wildtype (ND1-Arpp21) or mutant (ND1-Arpp21-shRes) expression vector together with either a control shRNA (shNeg) or an shRNA directed against *Arpp21*. Western blot showing efficacy of knockdown for wildtype but not mutant shRNA-resistant ARPP21 is shown. (**b**) Representative neuron after IUE with shArpp21 and ND1-Arpp21-shRes plasmids reconstructed after biocytin filling. (**c,d**) Co-electroporation of the shRNA-resistant NeuroD1::Arpp21 expression construct together with a shRNA construct against *Arpp21* partially rescues the phenotype of reduced dendritic arborization as shown by Sholl analysis. For the purpose of clarity the data are plotted with (**c**)

and without (**d**) the non-targeting shRNA control (shNeg). (**) $p < 0.01$, 2-way ANOVA with Bonferroni post-test. Data are presented as mean ± s.e.m. of n = 11 cells (shArpp21 and shArpp21+ND1-Arpp21) and n = 15 cells (shNeg). Scale bar: 50 µm. The lack of full rescue may be due to the earlier onset of expression of the Arpp21 shRNA relative to the neuron-specific NeuroD1 promoter used to express ARPP21.

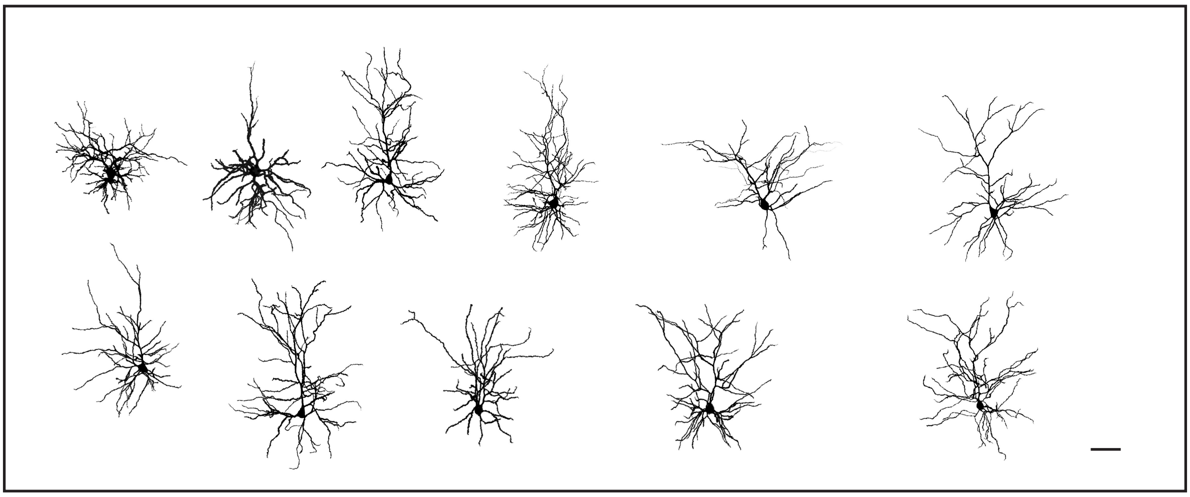

Supplementary Figure 17. Complete set of cortical neuron reconstructions from the *Arpp21* shRNA rescue experiment. Scale bar: 50 µm.

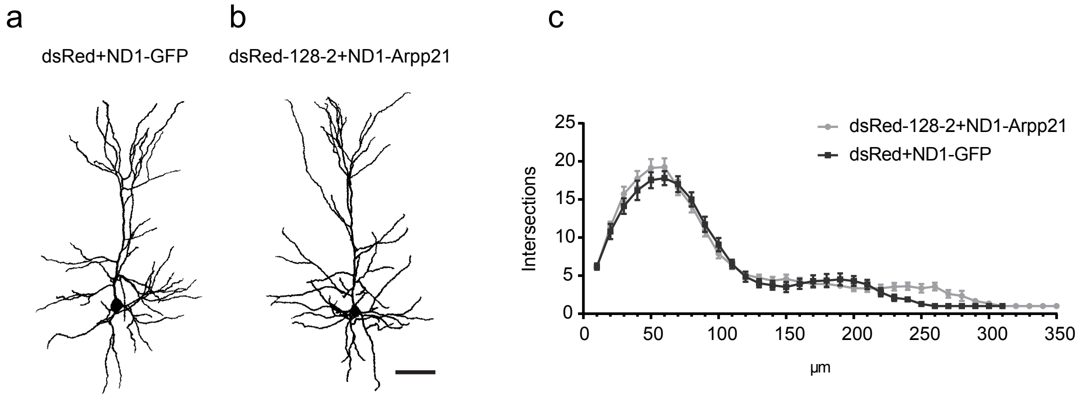

Supplementary Figure 18. Rescue of the *Arpp21* gain-of-function dendritic phenotype by miR-128 co-expression. Representative reconstructions of control neurons expressing dsRed and GFP (**a**) and neurons expressing miR-128 and *Arpp21* under the NeuroD1 promoter (**b**). (**c**) Sholl analysis of dsRed+ND1-GFP (black) and dsRed-128-2+ND1-Arpp21 (grey) expressing neurons after IUE shows no significant differences in the dendritic complexity between the two conditions. Data are presented as mean ± s.e.m. of n = 15 cells (dsRed+ND1-GFP) and n = 35 cells (dsRed-128-2+ND1-Arpp21). Scale bar: 50 µm.

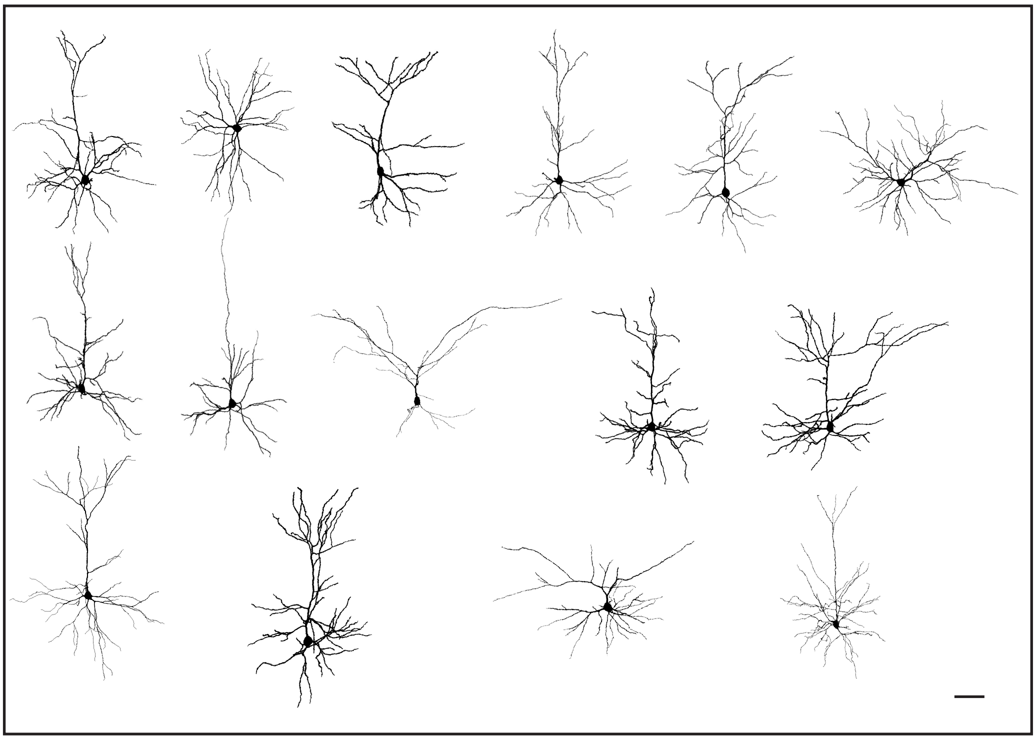

Supplementary Figure 19. Complete set of cortical neuron reconstructions from the dsRed+ND1-GFP condition. Scale bar: 50 µm.

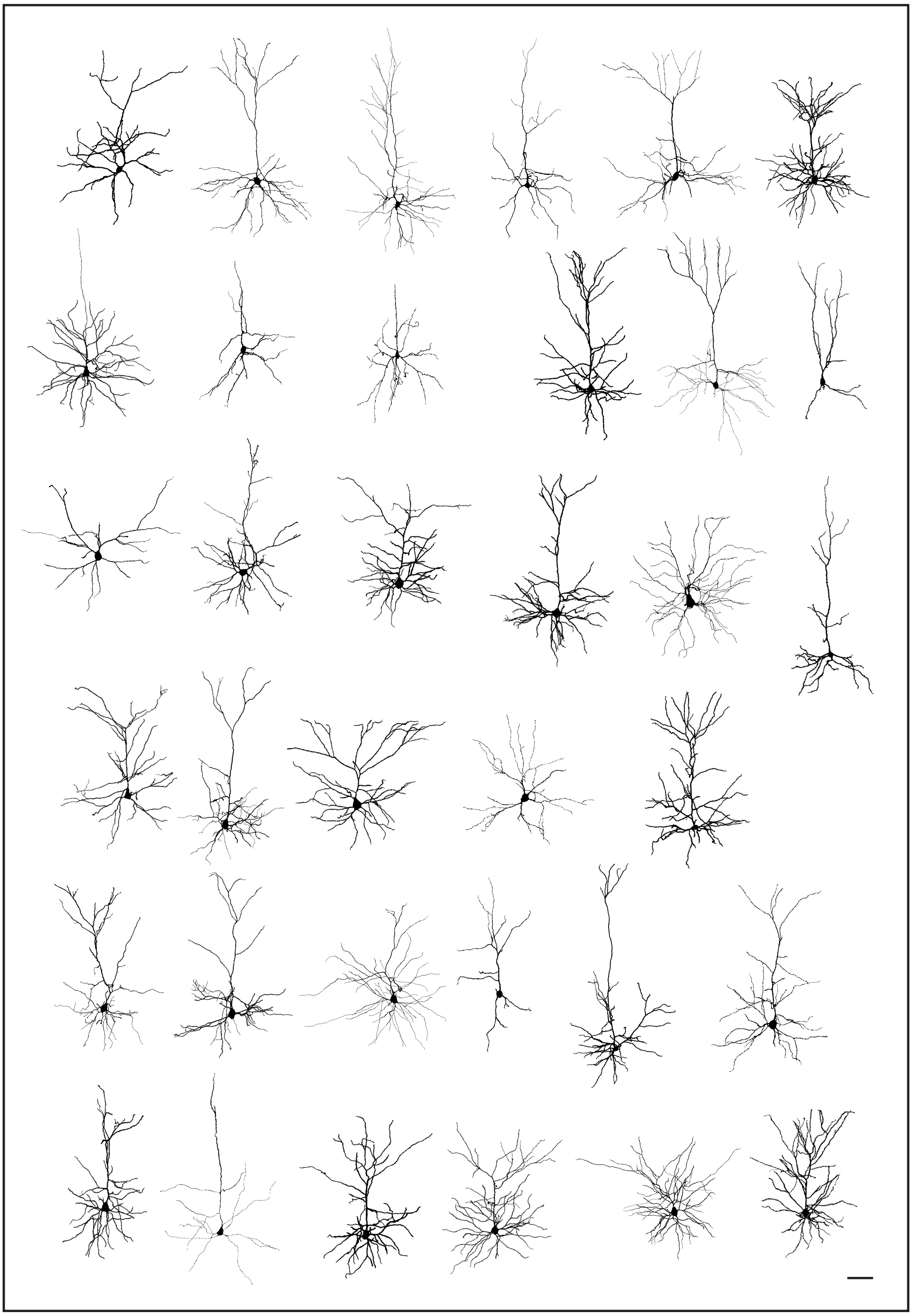

Supplementary Figure 20. Complete set of cortical neuron reconstructions from the dsRed-128-2+ND1-Arpp21 condition. Scale bar: 50 µm.

a

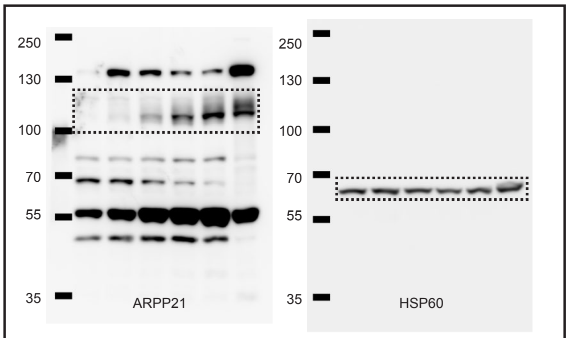

Related to Fig. 1e

b

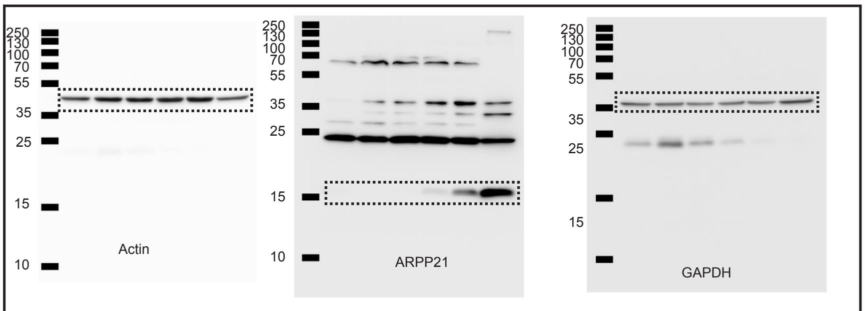

Related to Fig. 1f

c

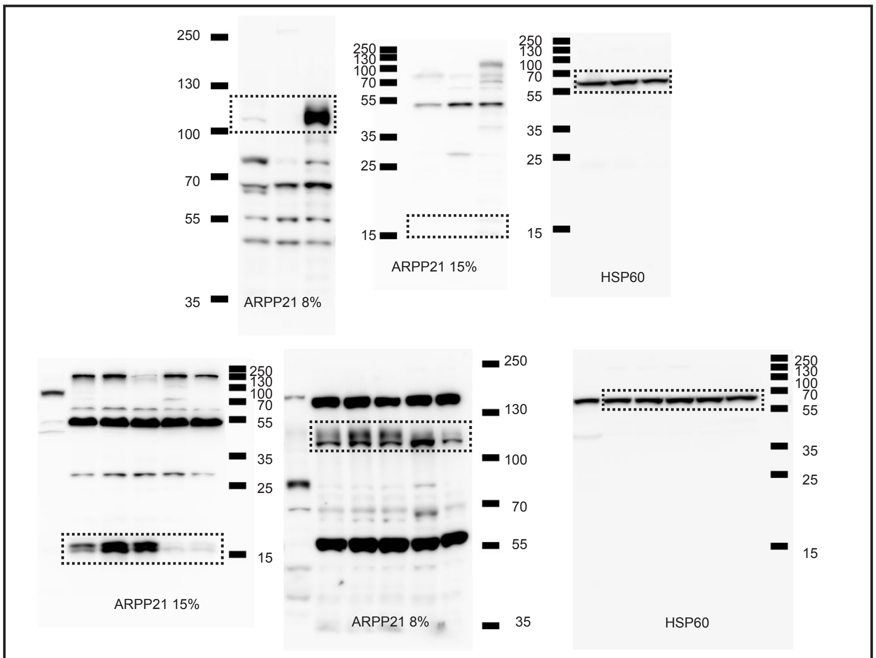

Related to Fig. 1g

d

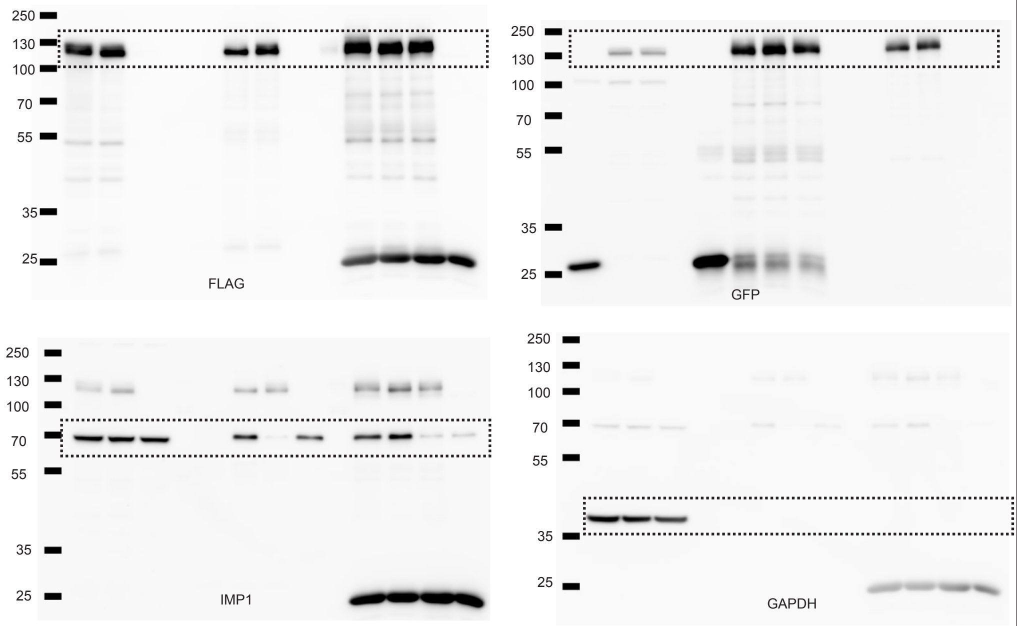

Related to Fig. 3f

e

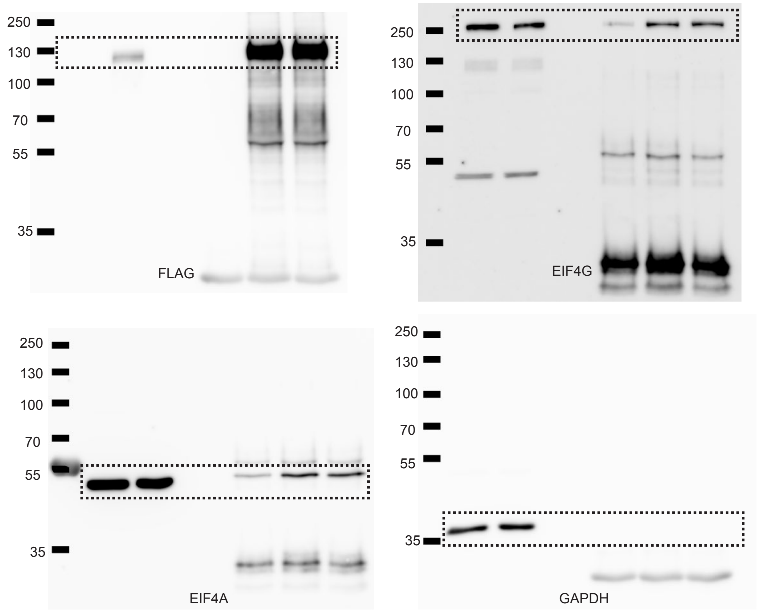

Related to Fig. 3g

f

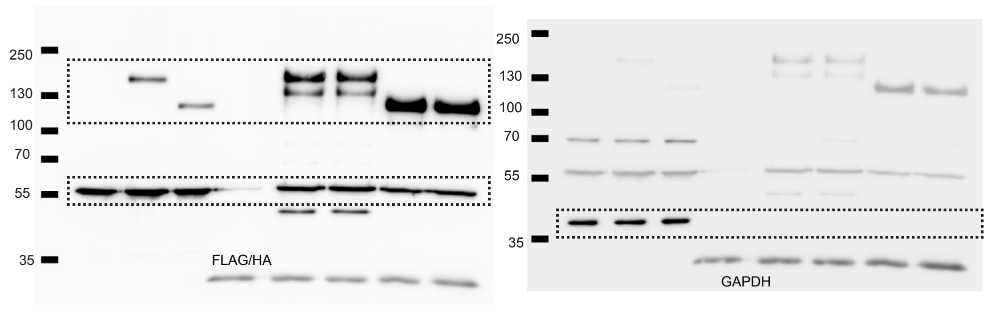

Related to Fig. 3h

g

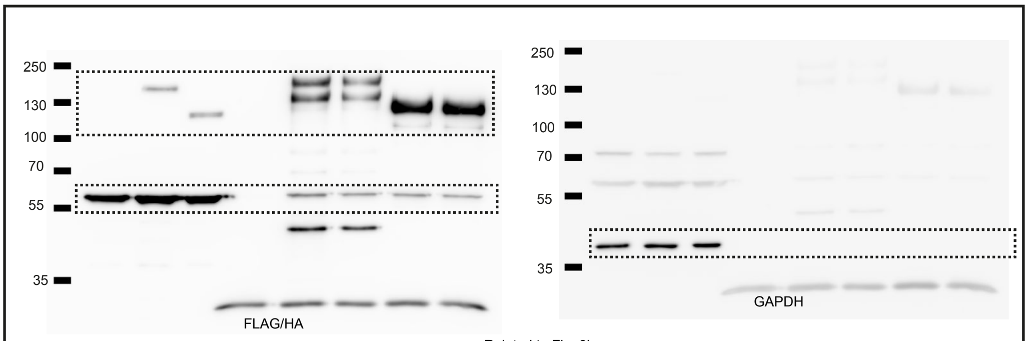

Related to Fig. 3i

h

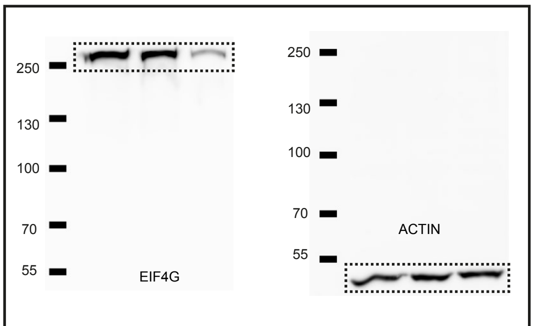

Related to Fig. 3j

i

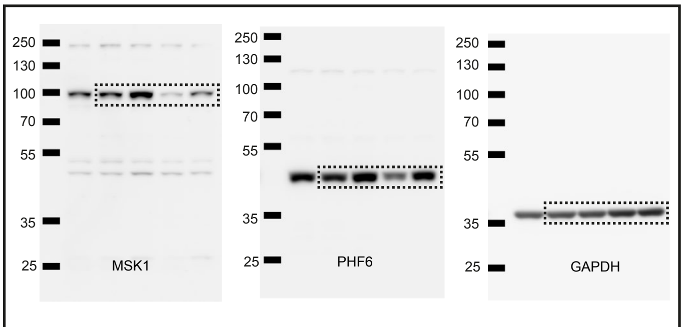

Related to Fig. 6f

j

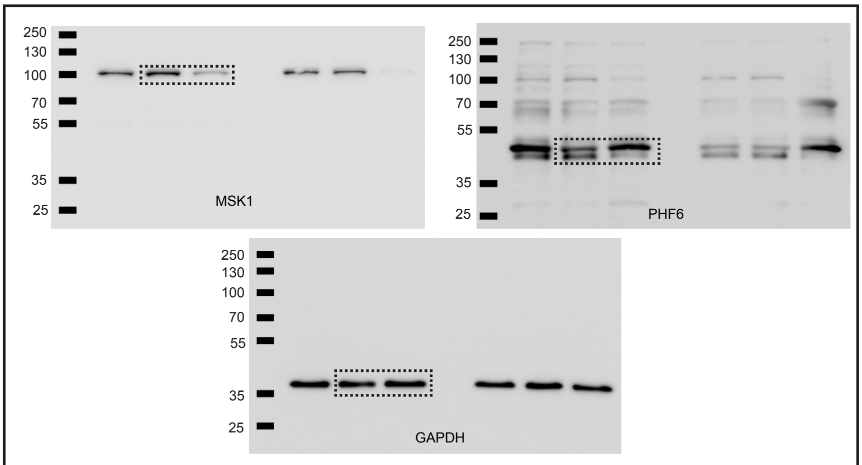

Related to Fig. 6i

k

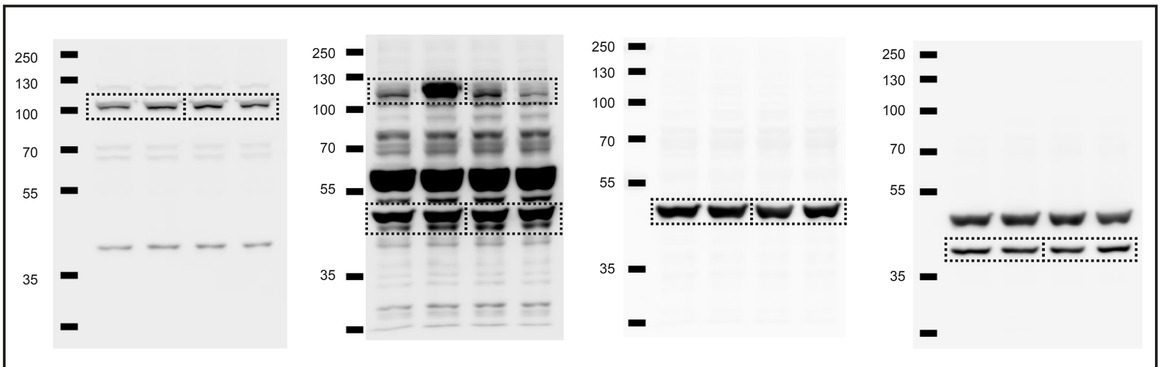

Related to Fig. 6k,l

l

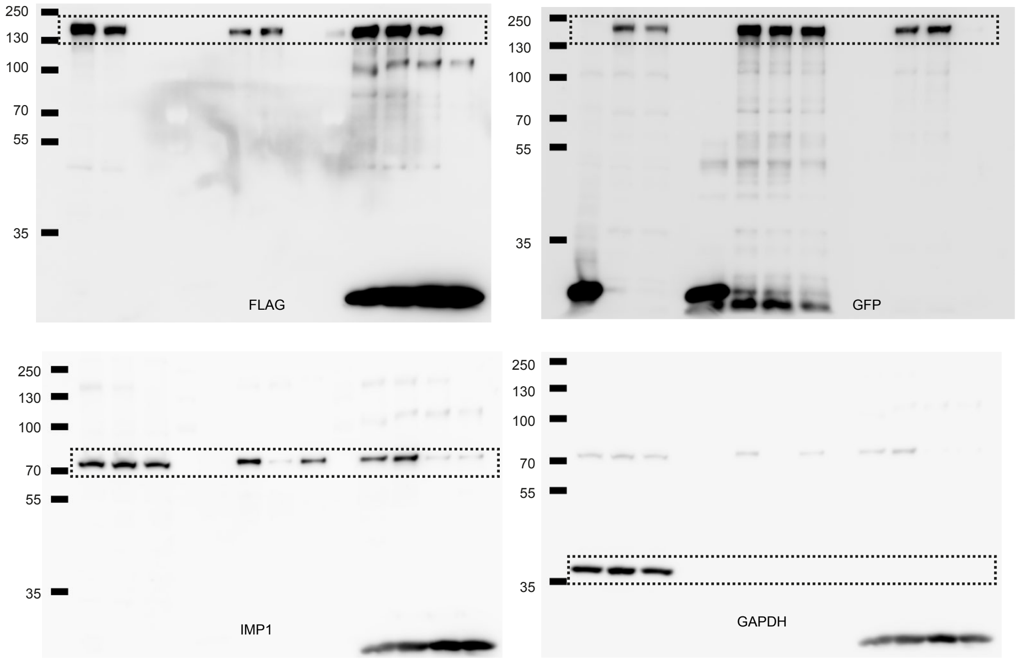

Related to Supplementary Fig. 4b

m

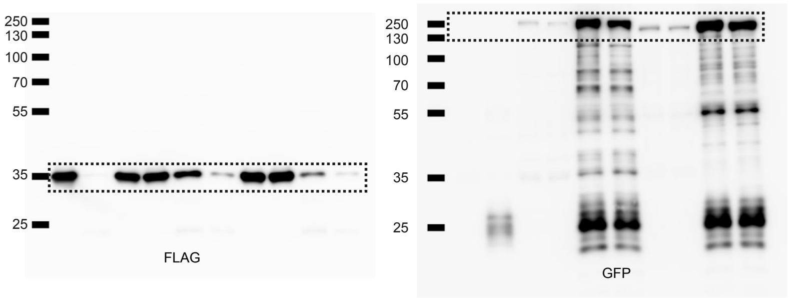

Related to Supplementary Fig. 4c

n

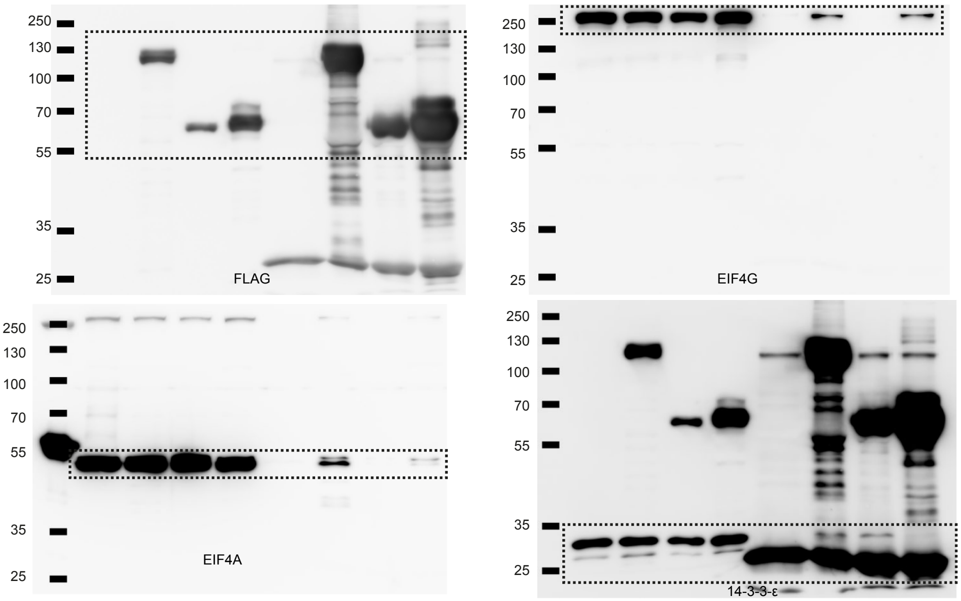

Related to Supplementary Fig. 4e

o

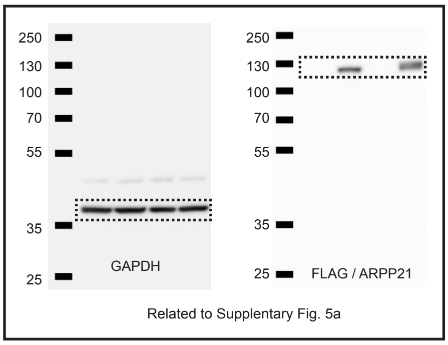

p

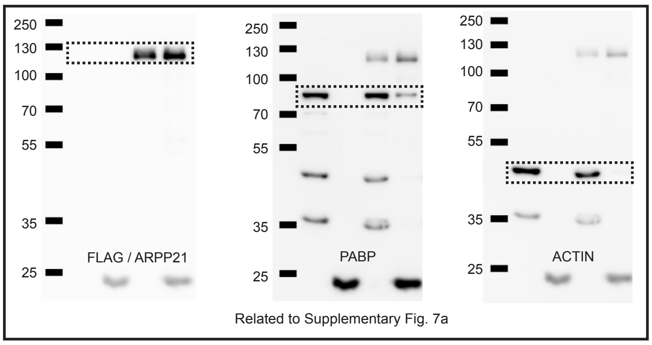

q

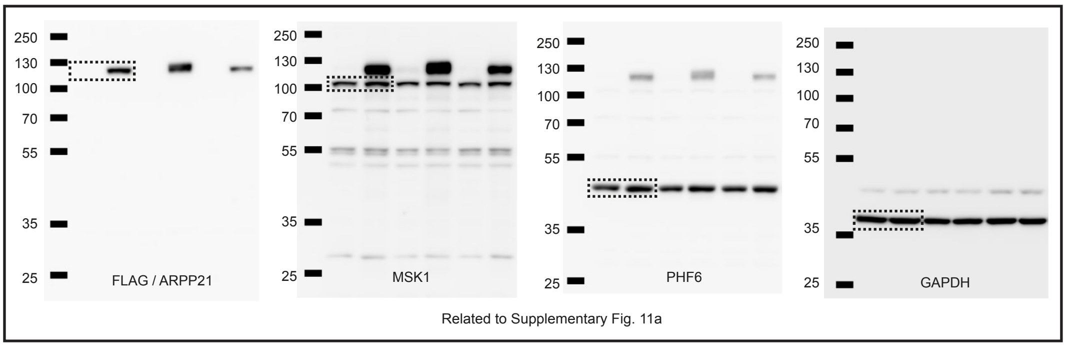

r

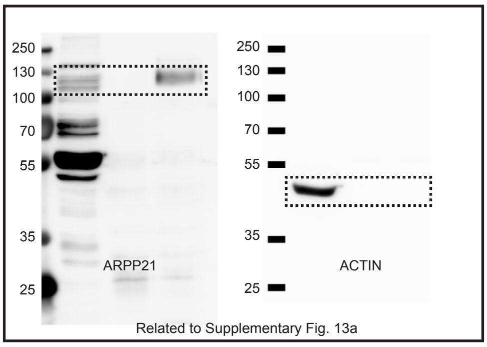

s

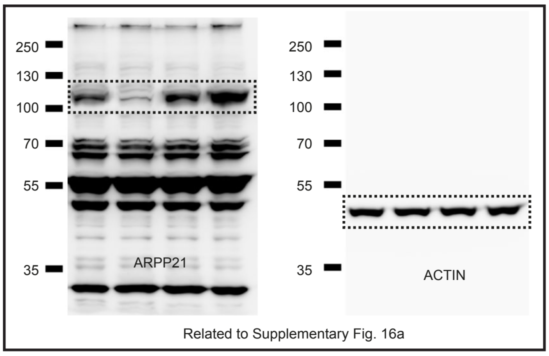

Supplementary Figure 21. Full images of cropped Western Blots. (**a**) Related to Fig. 1e. (**b**) Related to Fig. 1f. (**c**) Related to Fig. 1g. (**d**) Related to Fig. 3f. (**e**) Related to Fig. 3g. (**f**) Related to Fig. 3h. (**g**) Related to Fig. 3i. (**h**) Related to Fig. 3j. (**i**) Related to Fig. 6f. (**j**) Related to Fig. 6i. (**k**) Related to Fig. 6k,l. (**l**) Related to Supplementary Fig. 4b (**m**) Related to Supplementary Fig. 4c. (**n**) Related to Supplementary Fig. 4e. (**o**) Related to Supplementary Fig. 5a. (**p**) Related to Supplementary Fig. 7a. (**q**) Related to Supplementary Fig. 11a. (**r**) Related to Supplementary Fig. 13a. **(s)** Related to Supplementary Fig. 16a.

Supplementary Table 1a. Genomic localization of miR-128 among species.		
**Species**	**miRNA**	**Genomic localization**
Anolis carolinensis	aca-miR-128-1	Arpp21
	aca-miR-128-2	R3hdm1
Bos taurus	bta-miR-128-1	R3hdm1
	bta-miR-128-2	Arpp21
Canis familaris	cfa-miR-128-1	R3hdm1
	cfa-miR-128-2	Arpp21
Danio Rerio	dre-miR-128-1	R3hdm1
	dre-miR-128-2	Arpp21
Equus caballus	eca-miR-128-1	Arpp21
	eca-miR-128-2	R3hdm1
Gallus gallus	gga-miR-128-1	R3hdm1
	gga-miR-128-2	Arpp21
Gorilla gorilla	ggo-miR-128	R3hdm1
Homo sapiens	hsa-miR-128-1	R3hdm1
	hsa-miR-128-2	Arpp21
Monodelphis domestica	mdo-mir-128a	Arpp21
	mdo-mir-128b	R3hdm1
Macaca mulatta	mml-miR-128a	R3hdm1
	mml-miR-128b	Arpp21
Mus musculus	mmu-miR-128-1	R3hdm1
	mmu-miR-128-2	Arpp21
Ornithorhynchus anatinus	oan-mir-128-2	Arpp21
Oryzias latipes	ola-mir-128	Unc. gene with R3h domain
Petromyzon marinus	pma-mir-128-1	R3hdm1 1 of 2
	pma-mir-128-2	R3hdm1 2 of 2
Pongo pygmaeus	ppy-miR-128-1	R3hdm1
	ppy-miR-128-2	Arpp21
Pan troglodytes	ptr-mir-128-1	R3hdm1
	ptr-mir-128-2	Arpp21
Rattus norvegicus	rno-miR-128-1	R3hdm1
	rno-miR-128-2	Arpp21
Sarcophilus harrisii	sha-mir-128	R3hdm1
Sus scrofa	ssc-mir-128-1	R3hdm1
	ssc-mir-128-2	Arpp21
Taeniopygia guttata	tgu-mir-128-1	R3hdm1
	tgu-mir-128-2	Arpp21
Tetraodon nigroviridis	tni-mir-128-1	R3hdm1
	tni-mir-128-2	Arpp21
Xenopus tropicalis	xtr-mir-128-1	R3hdm1
	xtr-mir-128-2	Intergenic*
*No Arpp21 annotated. Short genome assembly surrounding miR-128-2.		

Supplementary Table 1b. Conservation of the miR-128 binding site in the Arpp21 3'UTR.	
Mouse	UACCACUGAAUACCACUGUGU
Human	UACCACUGAAUACCACUGUGU
Chimp	UACCACUGAAUACCACUGUGU
Rhesus	UACCACUGAAUACCACUGUGU
Squirrel	UACCACUGAACACCACUGUGU
Rat	UACCA-UGAAUACCACUGUGU
Rabbit	UACCACUGAAUACCACUGUGU
Pig	UACCACUGAAUACCACUGUGU
Cow	UACCACUGAAUACCACUGUGU
Cat	UACCACUGAACACCACUGUGU
Dog	UACCACUGAAUACCACUGUGU
Brownbat	UACCACUGAAUACCACUGUGU
Elephant	UACCACUGAAUACCACUGUGU
Opossum	---------------------
Macaw	UACCACUGAACACCACUGUGU
Chicken	UACCACUGAACACCACUGUGU
Lizard	UACCACUGAACACCACUGUGU
Xenopus	---------------------

Supplementary Table 1. Evolutionary conservation of miR-128 genomic localization and of the miR-128 seed match in the Arpp21 3'UTR. (a) Genomic localization of all miR-128 orthologs listed in miRBase release 21. **(b)** miR-128 binding site in the Arpp21 3'UTR and its conservation among species.

Supplementary Table 2. Proteins at least 2-fold enriched upon Arpp21 and R3hdm1 IP								
Protein names	Neg-MV-LFQ	Arpp21-MV-LFQ	R3hdm1-MV-LFQ	Neg Unique Peptides	Arpp21 Unique Peptides	R3hdm1 Unique Peptides	Arpp21/ Neg	R3hdm1/ Neg
Ubiquitin-associated domain-containing protein	0	465945	411690	0	2	2	∞	∞
Glycogen [starch] synthase, muscle	0	329055	7913350	0	2	8	∞	∞
Magnesium transporter protein 1	0	1223650	1129095	0	2	2	∞	∞
Structural maintenance of chromosomes protein	0	532800	759020	0	1	2	∞	∞
Polyadenylate-binding protein 4	0	481720	581700	3	6	7	∞	∞
Heat shock 70 kDa protein 6;Putative heat shock 70 kDa protein 7	0	3270200	3521050	7	11	11	∞	∞
Chaperone activity of bc1 complex-like,	0	813265	686375	0	3	3	∞	∞
DDB1- and CUL4-associated factor 7	0	494935	664250	0	1	1	∞	∞
Nuclear pore complex protein Nup155	0	980980	223790	0	3	2	∞	∞
Probable ATP-dependent RNA helicase DDX5	0	1261220	2100415	1	3	5	∞	∞
Sideroflexin-3	0	6614550	3915850	0	2	2	∞	∞
Flap endonuclease 1	0	379925	437765	0	1	1	∞	∞
Protein SEC13 homolog	0	209080	674350	0	1	2	∞	∞
NADH dehydrogenase [ubiquinone] 1 beta subcomplex subunit 9	0	266070	326890	0	2	2	∞	∞
14-3-3 protein zeta/delta;14-3-3 protein sigma	0	1555445	15054500	4	7	8	∞	∞
Pre-mRNA-processing factor 6	0	750000	867450	0	1	1	∞	∞
Zinc finger protein RFP	0	97555	3209650	0	2	7	∞	∞
Proteasome subunit beta type-1;Proteasome subunit beta type	0	341855	130335	0	1	1	∞	∞
Adenomatous polyposis coli protein	0	1666750	1336950	0	1	1	∞	∞
Dermcidin;Survival-promoting peptide;DCD-1	0	254690	352135	0	1	1	∞	∞
Transmembrane 9 superfamily member 3	0	431175	643290	1	2	2	∞	∞
FAST kinase domain-containing protein 5	0	1550400	2073250	0	2	2	∞	∞
E3 ubiquitin-protein ligase RNF138	0	1096020	954820	0	2	2	∞	∞
Regulator of nonsense transcripts 1	0	540700	484870	0	1	1	∞	∞
RNA-binding protein 14	0	46076000	50945000	0	19	21	∞	∞
cAMP-regulated phosphoprotein 21	0	1901300000	255305	0	18	2	∞	∞
DnaJ homolog subfamily B member 11	0	514605	778075	0	2	2	∞	∞
NADH dehydrogenase [ubiquinone] iron-sulfur protein 2, mitochondrial	213445	4015450	1362650	1	3	1	18,8	6,4
Centromere/kinetochore protein zw10 homolog	141185	2602350	929640	1	3	2	18,4	6,6
R3H domain-containing protein 1	88690	1628550	2788500000	2	7	38	18,4	31441,0
Heterogeneous nuclear ribonucleoprotein M	30332500	425170000	72635500	25	35	31	14,0	2,4
14-3-3 protein beta/alpha;14-3-3 protein beta/alpha, N-terminally processed	188290	2603200	7965050	4	7	11	13,8	42,3
Fibronectin type-III domain-containing protein	132605	1829400	1231050	1	2	2	13,8	9,3
Puromycin-sensitive aminopeptidase	119405	1504245	317975	1	3	2	12,6	2,7
Dolichol-phosphate mannosyltransferase	75640	878500	287215	1	2	1	11,6	3,8
Matrin-3	440625	4942100	1958500	2	6	4	11,2	4,4
PNKD	185930	2070200	1749150	2	2	2	11,1	9,4
Acylglycerol kinase, mitochondrial	394180	4353200	4330200	3	7	9	11,0	11,0
78 kDa glucose-regulated protein	28326500	300615000	666475000	27	45	51	10,6	23,5
NADH dehydrogenase [ubiquinone] iron-sulfur protein 3, mitochondrial	112575	1160300	963560	1	2	2	10,3	8,6
Protein TBRG4	60385	620505	486005	1	2	2	10,3	8,0
Tricarboxylate transport protein, mitochondrial	210705	2045850	575865	1	2	2	9,7	2,7
Serine/threonine-protein kinase OSR1	131590	1186500	812760	1	2	2	9,0	6,2
Serine/threonine-protein phosphatase PGAM5, mitochondrial	1889800	16614000	9739150	4	5	4	8,8	5,2
Solute carrier family 35 member E1	43505	382430	252994	1	1	2	8,8	5,8
Heat shock protein 105 kDa	317705	2605200	4532750	3	6	7	8,2	14,3
40S ribosomal protein S5;40S ribosomal protein S5, N-terminally processed	161485	1241800	1541090	1	2	3	7,7	9,5
NADH-ubiquinone oxidoreductase 75 kDa subunit, mitochondrial	219080	1615250	1086970	1	4	2	7,4	5,0
Mitochondrial import inner membrane translocase subunit TIM50	787065	5687150	4523750	2	3	4	7,2	5,7
Long-chain-fatty-acid--CoA ligase 3	2897410	19014000	9641700	8	12	9	6,6	3,3
ATP-binding cassette sub-family D member 3	140825	912400	337645	1	2	1	6,5	2,4
ATP-binding cassette sub-family A member 1	217590	1384650	18907500	1	1	2	6,4	86,9
Heat shock 70 kDa protein 1A/1B	81376000	510530000	583700000	39	40	41	6,3	7,2
DnaJ homolog subfamily B member 6	320030	2000550	1042230	2	2	2	6,3	3,3
39S ribosomal protein L17, mitochondrial	86190	530205	274060	1	2	2	6,2	3,2
Heat shock cognate 71 kDa protein	113325000	671680000	679975000	36	37	43	5,9	6,0
Tubulin alpha-1C chain	4279300	24133500	23563000	43	34	43	5,6	5,5
Probable ubiquitin carboxyl-terminal hydrolase FAF-X;Ubiquitin carboxyl-terminal hydrolase;Probable ubiquitin carboxyl-terminal	219295	1235490	559845	2	3	2	5,6	2,6

Ataxin-10	558750	3118200	2289850	1	2	2	5,6	4,1
Cytochrome b-c1 complex subunit 1,	2166265	11923000	5191500	8	10	9	5,5	2,4
Translational activator GCN1	690545	3795600	1991900	3	8	6	5,5	2,9
Dehydrogenase/reductase SDR family	244220	1267000	1626050	1	3	2	5,2	6,7
Up-regulated during skeletal muscle growth	651295	3296300	1967450	2	3	2	5,1	3,0
Stress-70 protein, mitochondrial	33195500	162935000	201175000	29	33	38	4,9	6,1
Sideroflexin-1	5719250	27871500	27847000	8	11	10	4,9	4,9
39S ribosomal protein L3, mitochondrial	371895	1812000	1369750	1	2	3	4,9	3,7
DnaJ homolog subfamily A member 3,	235960	1112950	997630	2	2	2	4,7	4,2
28S ribosomal protein S12, mitochondrial	453135	2079350	1315850	1	2	2	4,6	2,9
RNA-binding protein 33	712050	3254100	7164250	2	2	4	4,6	10,1
Replication factor C subunit 2	246755	1116085	774200	1	2	2	4,5	3,1
Protein phosphatase 1G	194945	869100	1172290	1	2	3	4,5	6,0
28S ribosomal protein S2, mitochondrial	509595	2224100	1891350	2	3	2	4,4	3,7
ATP synthase subunit g, mitochondrial	547880	2363950	3545250	3	3	5	4,3	6,5
Transportin-1;Transportin-2	721300	3004350	1974950	3	6	8	4,2	2,7
40S ribosomal protein S3	11442000	46709500	33062000	11	13	14	4,1	2,9
60S acidic ribosomal protein P0;60S acidic ribosomal protein P0-like	8722250	35460500	26058000	9	12	11	4,1	3,0
Bifunctional glutamate/proline--tRNA ligase;Glutamate--tRNA ligase;Proline--tRNA	6004950	23840000	12147000	13	24	20	4,0	2,0
Signal peptidase complex subunit 2	415045	1644100	1121580	2	2	2	4,0	2,7
60S ribosomal protein L11	12048000	46296500	32680000	4	4	4	3,8	2,7
ATPase family AAA domain-containing	48608	185650	605345	4	5	10	3,8	12,5
Sterol-4-alpha-carboxylate 3-dehydrogenase, decarboxylating	814615	3071650	3490150	2	1	2	3,8	4,3
Importin-7	2524550	9382250	5164150	11	9	7	3,7	2,0
Heat shock 70 kDa protein 4	408630,5	1508650	2814000	3	3	5	3,7	6,9
Sideroflexin-4	583000	2149300	3527600	2	2	4	3,7	6,1
NADH dehydrogenase [ubiquinone] 1 alpha subcomplex subunit 10, mitochondrial	2352500	8624650	13457500	8	7	11	3,7	5,7
Exportin-T	514400	1884150	2180400	2	3	4	3,7	4,2
Delta-1-pyrroline-5-carboxylate synthase;Glutamate 5-kinase;Gamma-	227735	829795	852720	2	3	4	3,6	3,7
28S ribosomal protein S21, mitochondrial	710570	2585450	4349050	2	2	3	3,6	6,1
Mitochondrial ribonuclease P protein 1	1917600	6861050	8017400	6	7	9	3,6	4,2
ATP synthase subunit f, mitochondrial	808850	2890300	2436150	4	4	4	3,6	3,0
A-kinase anchor protein 8	1034850	3667600	5314750	2	4	3	3,5	5,1
SAM domain and HD domain-containing	593155	2092750	1557800	2	3	2	3,5	2,6
Tubulin beta-6 chain	585580	2053550	5275050	18	17	19	3,5	9,0
39S ribosomal protein L16, mitochondrial	1064915	3691500	5463000	6	7	8	3,5	5,1
Heat shock protein beta-1	825385	2828000	1729650	2	3	4	3,4	2,1
Keratin, type I cytoskeletal 9	3962950	13524500	41087000	8	10	17	3,4	10,4
28S ribosomal protein S30, mitochondrial	418650	1416700	1677500	2	2	2	3,4	4,0
14-3-3 protein epsilon	6033850	20063500	154045000	12	11	20	3,3	25,5
Wings apart-like protein homolog	349795	1106090	1105520	2	3	4	3,2	3,2
Voltage-dependent anion-selective channel	2850950	8947250	5697400	5	7	6	3,1	2,0
Glutaryl-CoA dehydrogenase, mitochondrial	536100	1666850	1423650	1	4	3	3,1	2,7
Tubulin beta-4A chain	1004115	3111000	4372750	31	32	31	3,1	4,4
NADH dehydrogenase [ubiquinone] 1 alpha subcomplex subunit 5	256840	794650	1321200	1	1	2	3,1	5,1
28S ribosomal protein S29, mitochondrial	265290	817390	1234700	1	2	2	3,1	4,7
Oligosaccharyltransferase complex subunit	646870	1983000	1468050	2	2	2	3,1	2,3
A-kinase anchor protein 8-like	2236100	6852300	5319250	5	8	8	3,1	2,4
Condensin-2 complex subunit D3	525350	1576010	3235950	1	2	2	3,0	6,2
Exportin-5	1215170	3633600	2409700	3	3	3	3,0	2,0
39S ribosomal protein L43, mitochondrial	2047850	6058050	5163000	5	5	5	3,0	2,5
E3 ubiquitin-protein ligase RBX1	504645	1447065	1106150	2	2	2	2,9	2,2
RNA-binding protein 39	672680	1915850	1751850	4	6	6	2,8	2,6
Ornithine aminotransferase, mitochondrial;Ornithine aminotransferase, hepatic form;Ornithine aminotransferase,	1218500	3427300	2854050	3	5	4	2,8	2,3
Mitochondrial inner membrane protein	8817400	24758000	24197500	15	15	17	2,8	2,7
Lamin-B receptor	7529250	20786500	16018500	8	9	11	2,8	2,1
Signal peptidase complex subunit 3	448685	1218450	979220	2	2	2	2,7	2,2
28S ribosomal protein S22, mitochondrial	1743250	4673850	3791250	5	4	4	2,7	2,2
Pentatricopeptide repeat-containing protein 3, mitochondrial	2260600	6020150	6862250	6	7	9	2,7	3,0
39S ribosomal protein L23, mitochondrial	145060	383830	427890	2	1	2	2,6	2,9
Peptidyl-prolyl cis-trans isomerase FKBP8	507170	1337780	2577500	2	3	4	2,6	5,1
ATP synthase subunit gamma, mitochondrial;ATP synthase gamma chain	4338650	11358500	9705200	6	9	10	2,6	2,2
ATPase family AAA domain-containing	6715400	17094000	19891500	9	6	11	2,5	3,0
X-ray repair cross-complementing protein 6	662720	1648500	3222750	3	3	5	2,5	4,9

40S ribosomal protein S16	15838500	38786000	30986500	11	11	11	2,4	2,0
Calcium-binding mitochondrial carrier protein SCaMC-1	487740	1178550	3254250	2	2	4	2,4	6,7
Golgin subfamily A member 2	971930	2326440	7099600	4	5	9	2,4	7,3
Apoptosis-inducing factor 1, mitochondrial	17977000	42877500	52145500	12	16	15	2,4	2,9
General transcription factor II-I	560100	1323500	1694650	1	1	2	2,4	3,0
Lysophospholipid acyltransferase 7	276815	649490	807430	2	2	2	2,3	2,9
Casein kinase II subunit beta	2678850	6022500	10808250	3	3	5	2,2	4,0
Calcium-binding mitochondrial carrier	2453800	5460550	8856700	12	8	12	2,2	3,6
GTP-binding nuclear protein Ran	1983350	4411750	4224800	5	5	6	2,2	2,1
WD repeat-containing protein 18	823355	1826900	1711955	2	3	3	2,2	2,1
40S ribosomal protein S27;40S ribosomal protein S27-like	20215000	44677500	47471500	7	7	8	2,2	2,3
Histone H4	2684150	5830850	6668750	5	7	7	2,2	2,5
Proteasome subunit alpha type-5;Proteasome subunit alpha type	3682900	7894650	8130100	5	6	6	2,1	2,2
Polyadenylate-binding protein 1;Polyadenylate-binding protein 3	5647600	12029200	11603950	12	14	14	2,1	2,1
3-hydroxyacyl-CoA dehydratase 3	6288200	13343000	16673000	7	5	6	2,1	2,7
Dolichyl-diphosphooligosaccharide--protein glycosyltransferase 48 kDa subunit	2183000	4592500	5144100	4	3	5	2,1	2,4
Reticulocalbin-1	43378000	90473000	103126500	14	13	12	2,1	2,4
Prolyl 4-hydroxylase subunit alpha-1	12331000	25271500	49947500	18	18	19	2,0	4,1
Eukaryotic translation initiation factor 4	8676750	17711800	21311000	16	18	19	2,0	2,5
Translocase of inner mitochondrial membrane domain-containing protein 1	815665	1653500	2609950	2	2	2	2,0	3,2
40S ribosomal protein S4, X isoform;40S ribosomal protein S4, Y isoform 2	26147500	52497500	59894500	15	13	16	2,0	2,3
Keratin, type II cytoskeletal 1	5346450	10643250	55075000	11	11	27	2,0	10,3
Estradiol 17-beta-dehydrogenase 12	7164600	14205000	17531000	6	5	5	2,0	2,4
Eukaryotic initiation factor 4A-I;Eukaryotic initiation factor 4A-II	6554350	12912000	16082500	13	12	14	2,0	2,5
Probable ATP-dependent RNA helicase	3549800	6980300	9635050	3	5	5	2,0	2,7
DNA mismatch repair protein Msh2	1594350	3132700	3430250	3	3	4	2,0	2,2
Complement component 1 Q subcomponent-binding protein, mitochondrial	4205850	8252350	10846400	7	7	9	2,0	2,6
Forkhead box protein C1	183485	358405	877035	1	1	2	2,0	4,8

Supplementary Table 2. Proteins that are at least 2-fold enriched in eluates of ARPP21 and R3HDM1 immunoprecipitations compared to mock transfected control. Proteins are sorted with increasing enrichment after FLAG-ARPP21 IP over control IP. MV-LFQ: Mean value of label free quantification peptide intensities.

**Supplementary Table 3. Arpp21 and negative control iCLIP deep sequencing statistics.**								
**Experiment**	**Reads**	**Mapped total**	**Mapped unique**	**CL-events**	**Piranha peaks**	**% Mapped total**	**% Mapped unique**	**Average PCR duplicates**
Arpp1-C19	38'377'860	36'015'092	25'268'231	10'554'212	10'864	93,84%	70,16%	2,39
Arpp2-C19	35'887'127	34'015'615	24'169'014	8'422'782	9'550	94,79%	71,05%	2,87
Arpp3-C21	36'041'350	33'824'361	24'184'701	3'934'038	5'958	93,85%	71,50%	6,15
Arpp4-C21	22'470'660	21'220'345	15'021'941	1'559'663	3'486	94,44%	70,79%	9,63
**Total**	**132'776'997**	**125'075'413**	**88'643'887**	**24'470'695**		**94,20%**	**70,87%**	**3,62**
**Average/sample**	**33194249,3**	**31268853,3**	**22160971,8**	**6117673,75**				
**Experiment**	**Reads**	**Mapped total**	**Mapped unique**	**CL-events**	**Piranha peaks**	**% Mapped total**	**% Mapped unique**	**Average PCR duplicates**
Neg-C19	283'770	259'587	152'011	32'495	146	91,48%	58,56%	4,68

Supplementary Table 3. Read mapping statistics of all ARPP21 iCLIP experiments and the negative control iCLIP.

Supplementary Table 4. Genomic localizations of Arpp21 iCLIP peaks.		
	Hits	Fraction as %
rRNA	5	0,013344365
tRNA	89	0,237529691
snoRNA	1	0,002668873
snRNA	25	0,066721823
lincRNA	1181	3,151938936
Misc ncRNA	489	1,305078865
Pseudogene	60	0,160132376
Protcod AS	191	0,509754731
3'UTR	26342	70,30345085
5'UTR	604	1,611999253
Exon	186	0,496410366
Intron	3269	8,724545624
Antisense	603	1,60933038
Not annotated	4424	11,80709386
All	37469	100

Supplementary Table 4. Genomic localizations of ARPP21 iCLIP peaks.

Supplementary Table 5. 7mer A1 seed matches	
**miRNA**	**7mer-A1 seed**
miR-128:	ACUGUGA
miR-132:	ACUGUUA
miR-124:	UGCCUUA
miR-150:	UGGGAGA
miR-9:	CCAAAGA
miR-1:	CAUUCCA
miR-138:	ACCAGCA
let-7:	UACCUCA
miR-302:	GCACUUA
miR-137:	GCAAUAA
miR-125:	UCAGGGA

Supplementary Table 5. 7mer-A1 seed matches used for enrichment analysis among Arpp21 iCLIP targets.

Supplementary Material and Methods

Antibodies

ACTIN (ms): MAB1501, Millipore. ARPP21 (rb): immunopurified peptide antibody raised against aa 10-26 of murine ARPP21, produced at Pineda Antikoerper Service, Berlin, Germany. EIF3η (gt): sc-16377, Santa Cruz. EIF4A (rb): #2013, Cell Signaling Technologies. EIF4G (rb): #2498, Cell signaling. FLAG (ms): #1804, Sigma. FXR2 (rb): #7098, Cell Signaling Technologies. G3BP (ms): 611126, BD Bioscience. GAPDH (ms): MAB374, Milipore. GFP (rb): ab290, Abcam. HA (ms): 26183, Thermo Scientific. HSP60 (ms): sc-13115, Santa Cruz. IMP1 (ms): sc-166344, Santa Cruz. MSK1 (rb): #3489, Cell Signaling Technologies. PHF6 (rb): NB100-68261, Novus Biologicals.

RNA isolation, reverse transcription and quantitative real-time PCR

Total RNA including miRNAs was isolated with the miRNeasy kit from Qiagen, Hilden, Germany including the optional on column DNase digestion. miRNA levels were quantified using ThermoScientific's TaqMan miRNA assays using proprietary primers. miRNA gene expression was normalized to the *sno-135* RNA. Coding gene expression was quantified through reverse transcription by oligo-dT priming and qRT-PCR analysis using ThermoScientific's Maxima SYBR green mix following standard protocols. Coding gene expression was normalized to the *Oaz1* housekeeping gene. Primer sequences are listed in the supplement.

qRT-PCR primer

Intron-spanning primers for quantitative real-time PCR (qRT-PCR) were designed whenever possible with amplicon sizes ranging between 80 and 250 bp. Primer efficiencies (E = 10^(-1/slope) * 100) were determined by a 1:5 serial dilution series for every newly designed primer

pair and had to lie within 90 – 110 %. To assure the specificity of newly designed primer pairs, their amplicon size was checked by gel electrophoresis on a 2% agarose gel.

qPCR-mmu-Oaz1-f: agtcagcgggatcacagtctt

qPCR- mmu-Oaz1-r: aggaccctggtcttgtcgtta

qPCR- mmu-Arpp21-short-f: ggctctctcttgataaggctga

qPCR- mmu-Arpp21-short-r: acagtccagggtgcttagacac

qPCR- mmu-Arpp21-long-f: aacctcacagcagtaccgacc

qPCR- mmu-Arpp21-long-f: agacaagactggctggtaacctg

qPCR- mmu-R3hdm1-f: atcacaggagacccaggagaa

qPCR- mmu-R3hdm1-r: cttggctggaatctcttgataac

qPCR- mmu-Phf6-f: gtgagccccactgcatttct

qPCR- mmu-Phf6-r: cgctgtctcgtagaccctttt

qPCR- mmu-Msk1-f: tggtgctgacagatttcggtc

qPCR- mmu-Msk1-r: caactgccttgtcgtgtccc

qPCR-hsa-Oaz1-f: atgatcggctgaatgtaacagag

qPCR-hsa-Oaz1-r: ccagttaatgcgtttggcgtc

qPCR-hsa-Rpl27-f: gatcgccaagagatcaaagataa

qPCR-hsa-Rpl27-r: ctctgaagacatccttattgacg

qPCR-18SrRNA-f: gtaacccgttgaaccccatt

qPCR-18SrRNA-r: ccatccaatcggtagtagcg

qPCR-hsa-Gapdh-f: tgggctacactgagcaccag

qPCR-hsa-Gapdh-r: caaattcgttgtcataccaggaa

qPCR-hsa-Eif4g1-f: cgggtgttcgactggatagag

qPCR-hsa-Eif4g1-r: tccttctgctcgtcacacagg

qPCR-hsa-Msk1-f: agcaaccttccacgccttta

qPCR-hsa-Msk1-r: agggtctccgggttattgct

qPCR-hsa-Phf6-f: agaggcacgaagctgatgtg

qPCR-hsa-Phf6-r: cagtggtagtggtatgtcctgtg

qPCR-hsa-Creb1-f: catcatctgctcccaccgta

qPCR-hsa-Creb1-r: ctgaataactgatggctgggc

qPCR-hsa-Msi2-f: cccaccatgagttagattcca

qPCR-hsa-Msi2-r: cactactgtgttcgcagataacc

qPCR-hsa-Casc3-f: aacggtgagcggctaaacaa

qPCR-hsa-Casc3-r: taaaaccagcacgaccttcctt

qPCR-hsa-Upf1-f: gtgtagccaagaccagccagt

qPCR-hsa-Upf1-f: cgcaggcaggatcgtgtat

qPCR-GFP-f: tgctgcccgacaaccactac

qPCR-GFP-r: acgaactccagcaggaccat

ThermoScientific's TaqMan miRNA assays were used to quantify gene expression of: sno-135 (#001230), miR-128 (#002216) and miR-124 (#001182). Reverse transcription was accomplished with the TaqMan miRNA reverse transcription kit (4366596) and qPCR was performed with the Taqman Fast Advance Master Mix (4444557).

***In situ* hybridization**

Adult mouse brain *in situ* data for ARPP21 was taken from the Allen Mouse Brain Atlas [2] experiments: #79677353 (ARPP21-long) and #68148752 (ARPP21-short). To analyze the localization of RNA expression in developing mouse brain tissue, *in situ* hybridization was performed. Digoxigenin (Dig) labeled, *in vitro* transcribed probe was hybridized to endogenous transcript immobilized in fixed brain tissue and visualized by a colorimetric reaction using

alkaline-phosphatase coupled anti-Dig antibody. Probes were designed to span 500 - 800 bp of coding sequence or 3' UTR. GC content of probes was kept around 50% and low complexity regions were avoided. PCR from cDNA templates was performed with Taq polymerase to generate TA overhangs and purified PCR product was ligated directly with linearized pGEM-T vector. The generated probe vector was linearized and gel purified. Depending on the orientation of the cloned probe fragment, SP6 or T7 RNA polymerase was used together with Dig-labeled ribonucleotides to *in vitro* transcribe the antisense strand of the transcript to be analyzed. After *in vitro* transcription, the RNA probe was precipitated by EtOH and LiCl and stored at -20°C.

Arpp21-short-probe-f: GATCTGTCTGAGCCCAGAGGA

Arpp21-short-probe-r: TTCTCTGGGAGTCAGTCCACAC

Arpp21-long-probe-f: AGAGAAGAACTGGCTGCCCAG

Arpp21-long-probe-r: AGCCTGCGTTGCTCACGTT

Hybridization of the probe to embryonic mouse brain sections and colorimetric development was done as previously described [7].

SDS-PAGE and Western Blot analysis

10 - 30 µg of protein lysates were mixed with 4X Laemmli probe buffer and boiled for 5 min at 98 °C. Protein samples were size separated by Tris-glycine buffered SDS-PAGE with gels composed of 6%, pH 6.8 stacking gels and 10% or 12% pH 8.8 separation gels. After electrophoresis, gels were blotted to PVDF membranes and blocked for 1 hour in 5% skim milk in 1 x PBS with 0.1% Tween-20 (PBST), rinsed, and incubated with primary antibody diluted in 3% BSA in PBST overnight at 4°C. Membranes were washed with PBST and incubated with HRP-conjugated secondary antibody in 5% skim milk PBST for 1 h. After washing with PBST the

HRP signal was detected with Clarity ECL (BioRad) and an ImageQuant LAS4000 mini (GE Healthcare) CCD-camera system.

Cell culture and transfection

293T, HeLa, N2A and 293-TREx cells were grown in DMEM supplemented with 10% fetal bovine serum, 100 U/mL penicillin and 100 µg/mL streptomycin in an incubator with 5% CO_2 and 95% humidity at 37 °C. Cells were passaged by trypsination after reaching 80-90% of confluence. Cell line transfection of plasmid DNA and siRNAs was performed with Lipofectamine 2000, ThermoScientific. For large scale experiments in 293T cells, e.g. for immunoprecipitations, polyethylenimine (PEI) was used to transfect plasmid DNA. 2 µl of PEI solution (2mg/ml PEI, titrated to pH 7 with HCl) were used per µg of plasmid DNA, mixed with the DNA in an appropriate volume of OptiMEM medium and incubated for 20 min at room temperature. The DNA-PEI mixture was then pipetted onto cells.

Cell lines

Cell lines were obtained directly from source. HeLa and N2A cells were obtained from the Leibniz Institute DSMZ (German Collection of Microorganisms and Cell Cultures). HEK-293T cells were a gift from L. Schauenburg, Freie Universität Berlin. The parental 293T-based Dox-inducible cell line used for generation of the iArpp21 cells was a kind gift from M. Landthaler, Max-Delbrück-Zentrum Berlin. Source cells were certified mycoplasma-free, however, we cannot exclude that mycoplasma contamination occurred during routine cell culture.

Plasmid constructs and molecular cloning

All PCRs for molecular cloning, except for the *in situ* probe generation, were performed with the Phusion II hot start proof-reading polymerase (Thermo Scientific). All cloned plasmids were checked for correctness by Sanger sequencing at GATC, Konstanz, Germany.

Site-directed mutagenesis of miR-128 binding site in Arpp21 3'UTR

The miR-128 seed match in the *Arpp21* 3'UTR was mutated by PCR based site-directed mutagenesis following [8].

Arpp21-3UTR-mut-f: cctaccactgaataccacaacgtagattacaatatccctgattcgg

Arpp21-3UTR-mut-r: gatattgtaatctacgttgtggtattcagtggtaggagaaaatcttaccttgat

FLAG-ARPP21 and FLAG-R3HDM1 overexpression

ARPP21 coding sequence (CDS) was cloned in-frame into the p3XFLAG-CMV7.1 vector (Sigma) by PCR with the following primers from murine cDNA after restriction with EcoRI and BamHI. ARPP21 full length: tttgaattcaatgtctgagcaaggaggactg (forward), ataggatcctctcagaacttgacctgcca (reverse); Arpp21-R3H: full length forward and ataggatccttatatcctggtgctgctggtc (reverse); Arpp21-R3H+SUZ: full length forward and ataggatccctacgaatctgtgctgctccaag; Arpp21 SUZ+Cterm: atagaattcgaccagcagcaccaggata (forward) and full length reverse; Arpp21 Cterm: atagaattcttggagcagcacagattcgg and full length reverse.

R3hdm1 CDS was cloned was cloned in-frame into the p3XFLAG-CMV7.1 vector (Sigma) by PCR from murine cDNA with the following primers and restriction with EcoRI and BamHI. R3hdm1 full length: ttagaattcgctaatgaggatgtctgatattgt (forward) and ttaggatccttactgagaacttgccctttc (reverse); R3hdm1-R3H: full length forward and ttaggatcctcactggctatcatctttgtcaaag; R3hdm1-R3H+SUZ: full length forward and ataggatccctaagaaagagagcctgtagaggaccc. R3hdm1-Cterm: atagaattcttggagcagcacagattcgg (forward) and full length reverse.

GFP tethering reporter

A linker oligonucleotide composed of ggccgcaagcttcgtacgctcgagacgcgtg (forward) and aattcacgcgtctcgagcgtacgaagcttgc (reverse) was cloned via NotI and EcoRI in the 3'UTR of a modified peGFP-C1 backbone (pXhoI) from [9]. A fragment containing four copies of an MS2

binding site was inserted as a BsiWI and EcoRI fragment from plasmid pDR1 (unpublished lab collection). Further details are available upon request.

MS2 coat gene fusion contructs

N-terminal MS2 coat binding fusion constructs were generated by PCR amplification of the MS2 coat CDS using the forward primer atatgcggccgccggcgcttctaactttactcagt and the reverse primer tacacaattggcgtagatgccggagtttgcc using peGFP-C1-MS2-NLS as template. The PCR product was digested by NotI and MfeI prior to ligation with fragments encoding Arpp21 and R3hdm1 obtained from their respective p3XFLAG-CMV7.1 expression plasmids after digestion with NotI and EcoRI. To generate the MS2-Ago2 fusion construct, the MS2 coat gene was amplified by PCR with ataaagcttgccggcgcttctaactttactcag (forward) and agcaggatccggcgtagatgccggagtttgcc (reverse) primers and digested by HindIII and BamHI. The p3XFLAG-myc-CMV-24-Ago2 construct (Addgene #21538) was digested by HindIII and BamHI and ligated with the digested PCR product. Additional details are available upon request.

Interaction candidate expression

Candidate interactors that were validated by co-immunoprecipitation were cloned by PCR from murine cDNA into the p3XFLAG-CMV7.1 (Sigma) backbone. The Eif4a1 CDS was amplified with aacctgaattccatgtctgcgagtcaggattctc (forward) and aaccgtcgacccaggtcgcaggacagccc (reverse) primers and cloned via EcoRI and SalI. Eif4a2 CDS was amplified by PCR with tgacgtcgacatcatgtctggtggctccg (forward) and tgacggatcccagggattaaattaggtcagcca (reverse) primers and cloned via SalI and BamHI. 14-3-3-ζ CDS was amplified with tgacgaattctatggataaaaatgagctggtgc (forward) and tgacgtcgactgaggcagacaaaggttggaa (reverse) and cloned via EcoRI and SalI.

Doxycycline inducible ARPP21 expressing cell line

Arpp21 CDS was amplified by PCR with acgcgtcgacatgtctgagcaaggaggactgac (forward) and atagtttagcggccgctcagaacttgacctgccagcc (reverse) primers and cloned into the pENTR4 vector (ThermoScientific) via SalI and NotI. From this pENTR4-Arpp21 donor construct *Arpp21* was transferred by Gateway-Cloning to the pFRT-FLAG-HA-DEST recipient vector to generate pFRT-FLAG-HA-DEST-Arpp21. This vector was used for transfection and recombination with the Flp-In-TREx cell line following a published protocol [10].

3'UTR reporter assays

All 3'UTRs were amplified from murine cDNA or genomic DNA with the following primers and cloned into the 3'UTR of a modified peGFP-C1 backbone containing a multicloning site in the 3'UTR (pXhoI) [9]. The respective restriction enzymes used for each primer pair are indicated in the primer names.

Arpp21-NotI-f:	tttgcggccgccgcaggctggcaggtcaagttc
Arpp21-r-blunt:	aacagcacatggaaagagagacggaa
Casc3-EcoRI-f:	ttgggaattcgctgtctactactcagagggcttc
Casc3-BamHI-r:	gaacggatccctccaagtcacaatgctgatcac
Msi2-XhoI-f:	gaacctcgaggcttccattgccgtctcact
Msi2-BamHi-r:	gaacggatccgttgtacttcctacggctgctg
Msk1-HindIII-f:	ataaagcttcccttgcacctcagctccct
Msk1-BamHI-r:	ataggatccctggggatattttttgatactctgac
Upf1-EcoRI-f:	ttgggaattcggtagcggaggaagagctaag

Upf1-BamHI-r: gaacggatcccatgttttattggtgacaaaaagc

The Phf6 3’UTR GFP reporter construct has been previously described [11].

Poly-uridine motif deletion from Upf1-3’UTR reporter

A synthetic DNA fragment consisting of the murine *Upf1* 3’UTR deleted of the following bases was ordered at Integrated DNA Technologies:

tttttctggtttttcttttttttttcttttttttggttttttttttttgtttgtttgtttggtttttttttttttttcttttttggtttt

Arpp21 shRNA expression constructs

Oligonucleotides used to generate shRNA inserts were denatured at 95 °C and annealed by subsequent cooling to room temperature in oligo-annealing buffer (10mM Tris pH 7.5, 50mM NaCl, 1mM EDTA). The annealed double-stranded oligonucleotides possessed overhangs for direct ligation with HindIII and BglII digested pSuper-retro-GFP-neo vector. For efficient GFP reporter expression *in vivo* the shRNA expression cassette was subcloned from pSuper into a pCAG-GFP-shlink construct. This plasmid is based on a CAG-GFP backbone and harbors an additional multiple cloning site for shRNA sublconing (shLink) inserted via SalI and SpeI. Plasmid details are available upon request. Oligonucleotide sequences used follow, the sense strand of the shRNA is underlined.

shRNA-Neg (f): gatccccttctccgaacgtgtcacgtttcaagagaacgtgacacgttcggagaattttta

shRNA-Neg(r): agcttaaaaattctccgaacgtgtcacgttctcttgaaacgtgacacgttcggagaaggg

shRNA-Arpp21 (f): gatccccgcctattcagagaatggaattcaagagattccattctctgaataggcttttta

shRNA-Arpp21 (r): agcttaaaaagcctattcagagaatggaatctcttgaattccattctctgaataggcggg

shLink (f): tcgacgagctcgtttaaaccaattga

shLink (f): ctagtcaattggtttaaacgagctcg

Arpp21 in vivo overexpression construct

The *Arpp21* CDS was amplified by PCR with primers atcagctagcatgtctgagcaaggaggactgac (forward) and atctggaattctcagaacttgacctgccagc (reverse) introducing NheI and EcoRI restriction sites and cloned into pNeuroD-IRES-GFP (Addgene #61403).

Lentiviral miR-128 overexpression constructs

Oligonucleotides for miR-128 overexpression were annealed as described for the Arpp21sh RNA constructs and cloned into the pSuper-retro-GFP-neo vector. Subsequently the miR-128-2 shRNA expression cassette including the H1 promoter from the pSuper-retro-GFP-neo vector were subcloned into a lentiviral plasmid of the Charité Viral Core Facility (BL-360), that drives RFP reporter gene expression under a synapsin promoter.

Sh-miR-128-2-F:

gatcccggggggccgatgcactgtaagagagtgagtagcaggtctcacagtgaaccggtctcttttttttta

Sh-miR-128-2-R:
agcttaaaaaaaagagaccggttcactgtgagacctgctactcactctcttacagtgcatcggccccccggg

Lentiviral Arpp21 overexpression construct

Arpp21 CDS amplified with atcagctagcatgtctgagcaaggaggactgac (forward) and atctggaattctcagaacttgacctgccagc (reverse) primers and cloned in frame via NheI and EcoRI after a human synapsin promoter driven GFP with P2A peptide at its C-terminus (Charité Viral Core facility vector BL-140).

Lentiviral Arpp21 knockdown construct

The control and Arpp21 knockdown shRNA expression cassette including the H1 promoter from the pSuper-retro-GFP-neo vector, see above, were subcloned into a lentiviral plasmid of

the Charité Viral Core Facility (BL-360), that drives RFP reporter gene expression under the synapsin promoter.

Immunocytochemistry

Cultured cells for microscopy were seeded on gelatin-coated cover slips or poly-L-lysine in case of primary cortical neurons. Cells were washed once with PBS and fixed with 4% PFA at room temperature for 10 min. After washing three times with PBS, cells were blocked in PBSTT+ (1X PBS, 2% BSA, 2% Donkey Serum, 0.1% TritonX-100, 0.05% Tween 20) for 2 h at room temperature. Cover slips with cells were stained with primary antibody in PBSTT+ overnight at 4 °C. After three washes in PBS the cover slips were incubated with secondary antibody diluted 1:500 - 1:250 in PBSTT+ for 1 h at room temperature. For nuclear staining, the secondary antibody dilution was supplemented with Draq5 (diluted 1:1000) dye. Cells were washed three more times in PBS and mounted.

Immunoprecipitation

Protein A/G agarose beads were washed twice in CoIP buffer (20mM Tris HCl pH 7.4, 150mM NaCl, 1% NP-40, 2mM EDTA, 10mM NaF, 1mM Na_3VO_4, 1X Proteinase inhibitor cocktail) and then incubated with 4-8 µg of antibody for 1 h at 4 °C in CoIP buffer. After incubation and three washing steps in CoIP buffer the beads were ready for use. For FLAG immunoprecipitation, FLAG-conjugated Agarose beads (A2220-1ML, Sigma) were used. Cells for immunoprecipitation were put on ice and washed twice with ice-cold PBS, scraped off in PBS with a rubber spatula and spun at 500 g for 3 min at 4 °C. The cell pellet was resuspended in 1 ml of ice-cold CoIP buffer. Cell lysates were incubated for 15 min on ice. The optional RNase treatment was performed by the addition of RNase A to a final concentration of 5 µg/ml and incubation for 4 min at 37 °C. Lysates were centrifuged 20 min at 14 000 g, 4 °C. 50 µl of supernatant were removed as input control and the remaining lysate was mixed with washed,

antibody-primed beads. Beads and lysates were incubated with end over end mixing for 2 h at 4 °C. Following the incubation at least four washing steps with CoIP buffer were performed. For elution of bound proteins 50 µl of 2X Laemmli buffer were added to the beads and incubated for 5 min at 98 °C.

Mass spectrometry analysis of host protein interactors

Immunoprecipitations for mass spectrometry were performed with one additional last washing step in pre-urea wash buffer (50mM Tris pH 8.5, 1mM EGTA, 75mM KCl). For elution, urea lysis buffer (6M Urea, 2M thiourea, 10mM HEPES pH 8.0) was added to the beads and incubated with shaking for 30 min at room temperature. The eluates were reduced by addition of DTT and subsequently alkylated by the addition of iodoacetamide. Eluted proteins were digested in two steps. First, LysC endopeptidase was used for digestion under denaturing conditions. Second, the samples were diluted with 50mM Ammoniumbicarbonate and trypsin was used for a second digestion under native conditions. Buffer exchange and desalting of peptides was accomplished by stop-and-go-extraction tips [12]. Peptides were separated via a Dionex Ultimate 3000 nano-HPLC device and measurement was performed on a LTQ Orbitrap Velos mass spectrometer. MS and MS/MS data were analyzed with the MaxQuant software and label free quantification.

siRNAs:

siRNA	*Catalogue number*	*Target sequence*	*Company*
Ctrl siRNA	4390843	Proprietary to Ambion	Ambion
EIF4G	s4585	cauucgucgcugaacagaa	Ambion

iCLIP

iCLIP (individual-nucleotide resolution cross-linking and immunoprecipitation) was performed with doxycycline inducible 293-TREx cells expressing HA-tagged full length ARPP21 (iARPP21

cells). The improved iCLIP protocol was performed essentially as described [13]. The following changes to the original protocol were made. Instead of a pre-adenlyated 3'-linker DNA oligonucleotide, linker adenylation was carried out enzymatically with the 5' DNA Adenylation Kit (E2610S) from New England Biolabs, following the manufacturer's instructions. The adenylated linker was purified using the Qiagen Nucleotide Removal Kit to remove ATP and ligase. The concentration of the purified, adenylated linker was measured with a nanophotometer and the linker stored in aliquots at -20°C. Only the medium size fraction of cDNAs (30-100 bp) was used for deep sequencing. The size distribution of the final, amplified library was assessed with a Bioanalyzer (Agilent) and its concentration was measured with a Qubit fluorescence spectrometer (ThermoScientific). From these two values the molarity of every iCLIP replicate library was calculated and 8 pmol of each replicate was multiplexed in one deep sequencing run. A total of four independent ARPP21 iCLIP experiments from two different iArpp21 clones were multiplexed together with two negative control iCLIP in two independent deep sequencing runs. 100 bp single read sequencing in high output mode was performed with TruSeq cluster generation v3 and TruSeq 50 cycle chemistry v3 on an Illumina HiSeq2500 sequencing platform. Sequencing was performed at the genomics platform of the Berlin Institute for Medical Systems Biology at the Max-Delbrück-Center Berlin, Germany.

iCLIP data processing

iCLIP read processing, mapping and PCR duplicate removal were performed using a Galaxy workflow integrating pysam (v 0.7.7), samtools (v1.2) [14], bedtools (v2.19.1) [15], Trim Galore! (v0.4.1), cutadapt (v1.8) [16], Flexbar (v2.5) [17], Bowtie2 (v2.2.6) [18], and bctools (https://github.com/dmaticzka/bctools, v0.2.0).

Reads were mapped to hg19 with bowtie2 using preset "--very-sensitive". We restricted all downstream analysis to uniquely mapped reads (where bowtie2 could not identify multiple

distinct alignments as indicated by the XS:i flag). PCR-duplicates were merged according to UMIs and filtered for spurious crosslinking events as described [19]. Peaks were called using Piranha [20] based on the crosslinked nucleotides of the combined replicates employing a bin size of 5 nt.

For motif detection peak regions were extended by 10 nt in both directions, regions that overlapped or touched after this extension were merged. The ARPP21 binding motif was identified using GraphProt; training sets of bound and unbound sequences were prepared as previously described [21].

RNA-seq expression levels

Expression levels from GSM1334293 / SRR1176647 were determined using sailfish (v0.10.1) [22] for Ensembl gene annotation release 75.

GO and KEGG pathway analysis

The gene lists used for gene enrichment analyses can be found in Supplementary Table 5. RNA-Seq data from a public TREx-293T cell line [5] was used as background control for the analyses (Supplementary Table 5a). GO biological process analysis was performed with the DAVID bioinformatics analysis platform [23]. KEGG pathway enrichment analysis was carried out with the WEB-based GEne SeT AnaLysis Toolkit (WEBGESTALT) [24].

RNA immunoprecipitation

One 6 cm dish of 293T cells was transfected with FLAG-tagged ARPP21 and lysed 24 hours after transfection. RNA-immunoprecipitation (RIP) was performed with FLAG-conjugated agarose beads; a control RIP from cells not expressing FLAG-ARPP21 was performed in parallel. Cells were washed twice with ice-cold PBS, harvested by scraping in RNase-free PBS and centrifuged for 3 min, 500 g at 4 °C. The cell pellet was resuspended in 600 µl of RIP lysis buffer (0.50% NP-40, 50mM TrisHCl pH 7, 150mM KCl, 2mM EDTA pH 8, 10mM NaF, 0.5mM DTT, 1X

Proteinase inhibitor cocktail, 0.2mM Na_3VO_4, 100U/ml RNase inhibitor) and kept 15 min on ice. Nuclei were pelleted at 14000 g for 20 min at 4 °C and the supernatant transferred to a new reaction vessel. 60 µl of lysate were removed as protein input control. 100 µl were taken out and mixed with 1 ml of TRIzol for RNA input control. Immunoprecipitation was performed for 90 min at 4 °C with end over end rolling. Beads were washed four times with RIP wash buffer (0.50% NP-40, 50mM TrisHCl pH 7, 150mM KCl, 10mM NaF, 0.5mM DTT, 0.2mM Na_3VO_4) and transferred in the last wash step to a new reaction vessel. In the last washing step 10% of the beads were separated and eluted for SDS-PAGE with 2X Laemmli buffer after boiling at 98 °C for 5 min. The remaining 90% were eluted by the addition of 1 ml TRIzol and RNA was prepared following the manufacturer's protocol. The RNA pellet was resuspended in 20 µl of mQII water. 50% of the isolated RNA was taken for further steps, the remaining 50% of RNA was stored at -80 °C. Eluate and input RNA were digested in 15 µl volume with 1.5 µl 10X DNase Buffer and 1 µl of RQ1 RNase-Free DNase (Promega). The reaction was incubated 30 min at 37 °C followed by the addition of 1.5 µl of DNase Stop solution and heat inactivation 10 min at 65 °C. DNase digested RNA was reverse transcribed in 20 µl with oligoDT priming and qRT-PCR was performed from the cDNA samples (see section qRT-PCR for details).

Synthetic miRNAs:

The following synthetic miRNAs and miRNA controls from ThermoScientific were used: miR-Neg#1 (AM17110) and miR-128 (PM11746).

Statistical analysis:

Statistical analysis was performed with GraphPad Prism 7. If not further specified, n-numbers represent independent experiments. For IUE, cells from at least three different animals were used for reconstruction and statistical analysis. For IUE analysis no

randomization was performed, neuron reconstruction was performed blinded. Sample size of the experiments was estimated according to a comparable previous study[10].

Supplementary References

1. Waterhouse AM, Procter JB, Martin DM, Clamp M, Barton GJ. Jalview Version 2--a multiple sequence alignment editor and analysis workbench. *Bioinformatics* **25**, 1189-1191 (2009).

2. Lein ES*, et al.* Genome-wide atlas of gene expression in the adult mouse brain. *Nature* **445**, 168-176 (2007).

3. Bruno IG*, et al.* Identification of a microRNA that activates gene expression by repressing nonsense-mediated RNA decay. *Mol Cell* **42**, 500-510 (2011).

4. Lin Q*, et al.* The brain-specific microRNA miR-128b regulates the formation of fear-extinction memory. *Nat Neurosci* **14**, 1115-1117 (2011).

5. Rybak-Wolf A, Jens M, Murakawa Y, Herzog M, Landthaler M, Rajewsky N. A variety of dicer substrates in human and C. elegans. *Cell* **159**, 1153-1167 (2014).

6. Molyneaux BJ*, et al.* DeCoN: genome-wide analysis of in vivo transcriptional dynamics during pyramidal neuron fate selection in neocortex. *Neuron* **85**, 275-288 (2015).

7. Srivatsa S*, et al.* Unc5C and DCC act downstream of Ctip2 and Satb2 and contribute to corpus callosum formation. *Nat Commun* **5**, 3708 (2014).

8. Liu H, Naismith JH. An efficient one-step site-directed deletion, insertion, single and multiple-site plasmid mutagenesis protocol. *BMC Biotechnol* **8**, 91 (2008).

9. Rybak A*, et al.* A feedback loop comprising lin-28 and let-7 controls pre-let-7 maturation during neural stem-cell commitment. *Nat Cell Biol* **10**, 987-993 (2008).

10. Spitzer J, Landthaler M, Tuschl T. Rapid creation of stable mammalian cell lines for regulated expression of proteins using the Gateway(R) recombination cloning technology and Flp-In T-REx(R) lines. *Methods Enzymol* **529**, 99-124 (2013).

11. Franzoni E*, et al.* miR-128 regulates neuronal migration, outgrowth and intrinsic excitability via the intellectual disability gene Phf6. *Elife* **4**, (2015).

12. Rappsilber J, Mann M, Ishihama Y. Protocol for micro-purification, enrichment, pre-fractionation and storage of peptides for proteomics using StageTips. *Nat Protoc* **2**, 1896-1906 (2007).

13. Huppertz I, *et al.* iCLIP: protein-RNA interactions at nucleotide resolution. *Methods* **65**, 274-287 (2014).

14. Li H, Durbin R. Fast and accurate short read alignment with Burrows-Wheeler transform. *Bioinformatics* **25**, 1754-1760 (2009).

15. Quinlan AR, Hall IM. BEDTools: a flexible suite of utilities for comparing genomic features. *Bioinformatics* **26**, 841-842 (2010).

16. Martin M. Cutadapt removes adapter sequences from high-throughput sequencing reads. *EMBnetjournal* **v. 17**, p. pp. 10-12 (2011).

17. Dodt M, Roehr JT, Ahmed R, Dieterich C. FLEXBAR-Flexible Barcode and Adapter Processing for Next-Generation Sequencing Platforms. *Biology (Basel)* **1**, 895-905 (2012).

18. Langmead B, Salzberg SL. Fast gapped-read alignment with Bowtie 2. *Nat Methods* **9**, 357-359 (2012).

19. Ilik IA, *et al.* Tandem stem-loops in roX RNAs act together to mediate X chromosome dosage compensation in Drosophila. *Mol Cell* **51**, 156-173 (2013).

20. Uren PJ, *et al.* Site identification in high-throughput RNA-protein interaction data. *Bioinformatics* **28**, 3013-3020 (2012).

21. Maticzka D, Lange SJ, Costa F, Backofen R. GraphProt: modeling binding preferences of RNA-binding proteins. *Genome Biol* **15**, R17 (2014).

22. Patro R, Mount SM, Kingsford C. Sailfish enables alignment-free isoform quantification from RNA-seq reads using lightweight algorithms. *Nat Biotechnol* **32**, 462-464 (2014).

23. Huang da W, Sherman BT, Lempicki RA. Systematic and integrative analysis of large gene lists using DAVID bioinformatics resources. *Nat Protoc* **4**, 44-57 (2009).

24. Wang J, Duncan D, Shi Z, Zhang B. WEB-based GEne SeT AnaLysis Toolkit (WebGestalt): update 2013. *Nucleic Acids Res* **41**, W77-83 (2013).
