# Supplementary material for: The RNA-binding protein ARPP21 controls dendritic branching by functionally opposing the miRNA it hosts
Source: Nat Commun. 2018 Mar 26;9:1235. doi: 10.1038/s41467-018-03681-3 (PMC5964322; doi:10.1038/s41467-018-03681-3)
Supplement: Supplementary file 3 — Description of Additional Supplementary Files(PDF 173 kb) [file 41467_2018_3681_MOESM3_ESM.pdf]

Description of Additional Supplementary Files

File Name: Supplementary Data 1
Description: Excel spreadsheet listing mRNAs identified in the ARPP21 iCLIP and all transcripts detected in the public TREx-293 RNA-Seq dataset. The TREx-293 expression data was used as background control for the KEGG pathway enrichment analysis and for the Supplementary Data 2.

File Name: Supplementary Data 2
Description: Excel spreadsheet listing conserved miR-128 target mRNA predictions from TargetsScan 7.1. Predicted genes that were not expressed in the TREx-293 cells (see Supplementary Data 1) are marked in column three.

File Name: Supplementary Data 3
Description: Excel spreadsheet showing the intersection of ARPP21 iCLIP targets and predicted miR-128 target mRNAs from TargetScan7.1.

File Name: Supplementary Data 4
Description: Excel spreadsheet listing transcripts not bound by ARPP21 that are expressed in TREx-293 cells with a TPM value above the lowest TPM of all ARPP21 iCLIP targets.

File Name: Supplementary Data 5
Description: Excel spreadsheet listing transcripts bound by ARPP21 with their respective CL-events and TPM.
